# Comprehensive analysis of a novel mouse model of the 22q11.2 deletion syndrome: a model with the most common 3.0-Mb deletion at the human 22q11.2 locus

**DOI:** 10.1038/s41398-020-0723-z

**Published:** 2020-02-05

**Authors:** Ryo Saito, Michinori Koebis, Taku Nagai, Kimiko Shimizu, Jingzhu Liao, Bolati Wulaer, Yuki Sugaya, Kenichiro Nagahama, Naofumi Uesaka, Itaru Kushima, Daisuke Mori, Kazuaki Maruyama, Kazuki Nakao, Hiroki Kurihara, Kiyofumi Yamada, Masanobu Kano, Yoshitaka Fukada, Norio Ozaki, Atsu Aiba

**Affiliations:** 1 Laboratory of Animal Resources, Center for Disease Biology and Integrative Medicine, Graduate School of Medicine, The University of Tokyo, Tokyo, Japan; 2 Department of Biological Sciences, School of Science, The University of Tokyo, Tokyo, Japan; 3 Department of Neuropsychopharmacology and Hospital Pharmacy, Nagoya University Graduate School of Medicine, Nagoya, Aichi Japan; 4 Department of Neurophysiology, Graduate School of Medicine, The University of Tokyo, Tokyo, Japan; 5 International Research Center for Neurointelligence (WPI-IRCN), The University of Tokyo Institutes for Advanced study (UTIAS), The University of Tokyo, Tokyo, Japan; 6 Department of Psychiatry, Nagoya University Graduate School of Medicine, Nagoya, Aichi Japan; 7 Medical Genomics Center, Nagoya University Hospital, Nagoya, Aichi Japan; 8 Department of Physiological Chemistry and Metabolism, Graduate School of Medicine, The University of Tokyo, Tokyo, Japan

**Keywords:** Molecular neuroscience, Psychiatric disorders

## Abstract

The 22q11.2 deletion syndrome (22q11.2DS) is associated with an increased risk for psychiatric disorders. Although most of the 22q11.2DS patients have a 3.0-Mb deletion, existing mouse models only mimic a minor mutation of 22q11.2DS, a 1.5-Mb deletion. The role of the genes existing outside the 1.5-Mb deletion in psychiatric symptoms of 22q11.2DS is unclear. In this study, we generated a mouse model that reproduced the 3.0-Mb deletion of the 22q11.2DS (*Del(3.0* *Mb)/* *+*) using the CRISPR/Cas9 system. Ethological and physiological phenotypes of adult male mutants were comprehensively evaluated by visual-evoked potentials, circadian behavioral rhythm, and a series of behavioral tests, such as measurement of locomotor activity, prepulse inhibition, fear-conditioning memory, and visual discrimination learning. As a result, *Del(3.0* *Mb)/* *+* mice showed reduction of auditory prepulse inhibition and attenuated cue-dependent fear memory, which is consistent with the phenotypes of existing 22q11.2DS models. In addition, *Del(3.0* *Mb)/* *+* mice displayed an impaired early visual processing that is commonly seen in patients with schizophrenia. Meanwhile, unlike the existing models, *Del(3.0* *Mb)/* *+* mice exhibited hypoactivity over several behavioral tests, possibly reflecting the fatigability of 22q11.2DS patients. Lastly, *Del(3.0* *Mb)/* *+* mice displayed a faster adaptation to experimental jet lag as compared with wild-type mice. Our results support the validity of *Del(3.0* *Mb)/* *+* mice as a schizophrenia animal model and suggest that our mouse model is a useful resource to understand pathogenic mechanisms of schizophrenia and other psychiatric disorders associated with 22q11.2DS.

## Introduction

The 22q11.2 deletion syndrome (22q11.2DS) is the most common microdeletion syndrome in humans, and estimated to affect up to 1 in 4000 live births^1^. Individuals with this syndrome display multiple physical abnormalities; cardiac malformation is the most frequent symptom affecting ~80% of patients, followed by less frequent symptoms, such as velopharyngeal insufficiency, hypocalcemia, thymus hypoplasia, and immune deficiency. In addition, 22q11.2DS is known to increase the risk of developing a variety of psychiatric and developmental disorders, including schizophrenia, intellectual disability, autism spectrum disorder, attention deficit hyperactivity disorder (ADHD), early-onset Parkinson’s disease, and sleep behavior disorder^2–6^. The total penetrance for psychiatric disorders reaches 100% in 22q11.2DS^7^. Particularly, the risk of schizophrenia imposed by this deletion (odds ratio: 16.3–44.2) is higher than any other schizophrenia-associated single genetic variations that have been reported so far^8–10^. Therefore, studying 22q11.2DS will shed light on the pathogenesis of schizophrenia.

Most (~90%) of the 22q11.2DS patients have a 3.0-Mb deletion on chromosome 22q11.2^11–15^. Forty-five protein coding genes are located within the 3.0-Mb deleted region, and 37 of them are conserved in the mouse chromosome 16qA13, making it possible to generate a mouse model of 22q11.2DS. To date, four lines of 22q11.2DS mouse models have been generated and analyzed^16–19^. However, all of them mimicked a half of the 3.0-Mb deletion (termed 1.5-Mb deletion), which is found in only 7% of 22q11.2DS patients, and the 3.0-Mb deletion has never been introduced to model animals^20^. Given that psychiatric diseases are highly heritable, an animal model of 22q11.2DS that precisely mimics 3.0-Mb deletion is necessary to investigate the pathology of psychiatric disorders more accurately. Individuals with the other half of the 3.0-Mb deletion (termed 1.4-Mb deletion) were diagnosed as ADHD and anxiety disorder^11^. Therefore, it is highly likely that genes responsible for the development of psychiatric disorders exist in the 1.4-Mb deleted region.

To this end, we generated a mouse model that reproduced the most common 3.0-Mb deletion in 22q11.2DS and characterized its phenotypes with multifaceted analyses related to psychiatric disorders. This mouse model displayed several phenotypes that reproduce human schizophrenia symptoms, such as reduction of auditory-dependent sensorimotor gating and attenuation of visual-evoked potential. This mouse model is expected to be a useful tool to decipher the pathogenic mechanisms of schizophrenia as well as 22q11.2DS.

## Materials and methods

### Animals

Animals were housed under a 12 h light/dark cycle (light on at 08:00, off at 20:00), with free access to food and water, at temperature maintained at 23 ± 1°C with a humidity of 50 ± 10%. The animal experiments were approved by the Institutional Animal Care and Use Committee of The University of Tokyo and the ethics committee of Nagoya University, and conducted in accordance with the guidelines of The University of Tokyo and Nagoya University.

### Generation of *Del(3.0Mb)/+* mice

To generate *Del(3.0* *Mb)/* *+* mice, we carried out CRISPR/Cas9-mediated genome editing by injected four single-guide RNAs (sgRNAs) and a single-stranded oligodeoxyribonucleotide (ssODN) into C57BL/6N mouse zygotes, as previously described^21^. The protospacer sequences of sgRNAs are listed in Supplementary Table 1. Deletion was confirmed by PCR, DNA sequencing, and array comparative genomic hybridization analysis. We obtained four founder mice with a desired mutation and found that half of them (two out of four) transmitted the deletion to the next generation (N1). We chose one founder female and used this line for this study. We crossed the *Del(3.0* *Mb)/* *+* male offspring (N1) of chosen founder female with C57BL/6N females. We obtained *Del(3.0* *Mb)/* *+* pups (N2) with desired deletion successfully. After N2 generation, we have maintained *Del(3.0* *Mb)/* *+* mice as a hemizygous line by crossing C57BL/6N females with hemizygous *Del(3.0* *Mb)/* *+* males. We used N2 mice in histological experiments, and N3–5 mice in behavioral experiments.

### RNA expression analysis

The total RNA was isolated from hippocampi or frontal cortexes of *Del(3.0* *Mb)/* *+* male (*n* = 5) and WT littermate male (*n* = 5) using the miRNeasy Mini Kit (QIAGEN, MD, USA). The details of mRNA and miRNA microarray analysis and quantitative RT-PCR are available in Supplementary Methods. The sequences of the quantitative RT-PCR primers are listed in Supplementary Table 2.

### Behavioral tests

*Del(3.0* *Mb)/* *+* and WT control littermates were obtained by in vitro fertilization between C57BL/6N eggs and *Del(3.0* *Mb)/* *+* sperm. One cohort of mice (WT, *n* = 13; *Del(3.0* *Mb)/* *+* , *n* = 9) was used for open-field test, visual PPI, five-trial social memory test, and fear-conditioning test. A second cohort of mice (WT, *n* = 11; *Del(3.0* *Mb)/* *+* , *n* = 11) was used for fear-conditioning test for additional trial to confirm the reproducibility of the results obtained from first trial. In cue-dependent fear-conditioning tests, we found significant difference in both the first cohort and the combined cohort. A third cohort of mice (WT, *n* = 15; *Del(3.0* *Mb)/* *+* , *n* = 15) was used for Y-maze, elevated-plus maze, locomotor activity, three-chamber test, auditory PPI, and rotarod test. A fourth cohort of mice (WT, *n* = 7; *Del(3.0* *Mb)/* *+* , *n* = 6) was used for visual discrimination task and reversal learning. All behavioral tests were carried out with male mice at the age of 2–5 months. Prior to behavioral tests, mice were placed in the testing room for at least 1 h to acclimate to the experimental environments. The experimenter was blind to genotype throughout the experimental procedures.

### Visual-evoked potentials

Visual-evoked potentials were recorded from *Del(3.0* *Mb)/* *+* (*n* = 7) and WT (*n* = 8) mice at the age of 11 weeks. A recording electrode of a stainless-steel wire was implanted into left visual cortex (2.2 mm lateral, 4.0 mm posterior to bregma, 400 μm ventral to the dura mater). A gold plating pin positioned in the right frontal bone (0.5 mm lateral, 3.0 mm anterior to bregma) served as a reference electrode. Visual stimuli were generated and presented according to the previous report^22^. Briefly, we presented static full-field square-wave grating (100% contrast, 0.04 cycles/degree) for 500 ms, followed by an interstimulus interval of 1000 ms of mean luminescence gray screen. A session was composed of 600 stimuli and lasted 15 min. The recorded data were prescreened for excessive artifact (e.g., signal >5 SDs).

### Statistical analyses

The significance of differences (*p* < 0.05) was assessed using the two-tailed Welch’s *t* test for comparisons of two groups. In multiple comparisons, the significance of differences was evaluated using an analysis of variance (ANOVA) with two-way repeated measures and Sidak’s multiple comparison post hoc test. All data are expressed as means ± SEM. We decided the sampling size (*n*) according to the same type of experiments in the previous reports^18,19^. The detailed statistics are described in Supplementary Table 3.

Detailed information of materials and methods is provided in Supplementary Methods.

## Results

### Generation of 22q11.2DS mouse model with a 3.0-Mb deletion (*Del(3.0Mb)/+* ) using CRISPR/Cas9 system

**Fig. 1:**
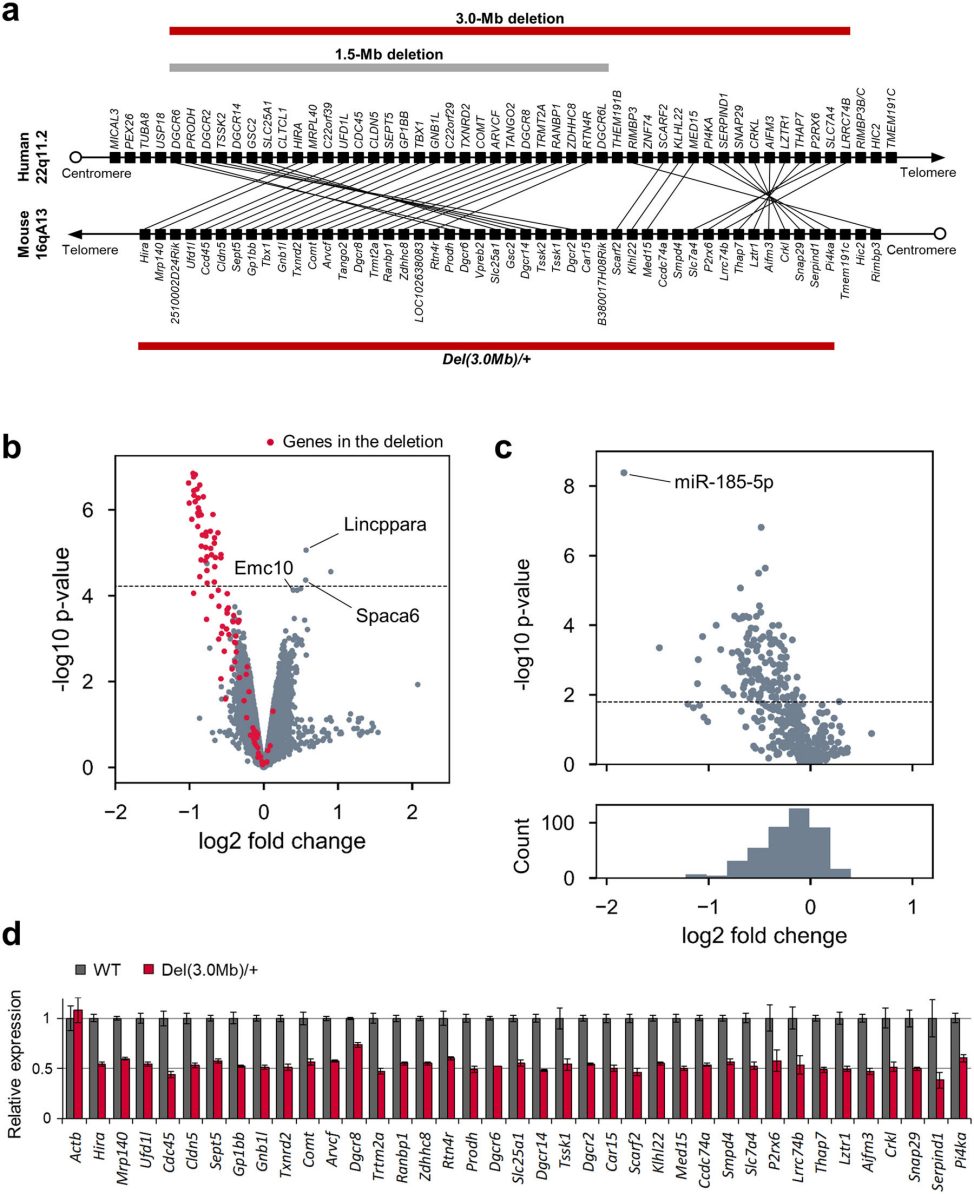
Generation and RNA expression of 22q11.2DS mouse model with a deletion syntenic to the human 3.0-Mb deletion. **a** Schematic diagram showing the human chromosome 22q11.2 region and the syntenic region of mouse chromosome 16qA13. Each black box represents one gene. Red horizontal bars indicate the hemizygous genomic deletion most frequently (~90%) found in human 22q11.2DS and syntenic mouse genomic region. A gray horizontal bar indicates the 1.5-Mb region less frequently (~7%) affected in 22q11.2DS. **b** Volcano plot of the mRNA microarray analysis data of the hippocampus. The *x*-axis shows log2 of the fold change (*Del(3.0* *Mb)/* *+* vs. WT). The genes in the deleted region are depicted in red. The horizontal dashed line indicates the *p*-value of FDR = 0.05. miRNA host genes (*Lincppara* and *Spaca6*) and miR-185 target gene *Emc10* were upregulated in *Del(3.0* *Mb)/* *+* hippocampus (see “Results”). **c** The microarray analysis of miRNA expression in the hippocampus. Top: volcano plot of the microarray analysis data. The horizontal dashed line indicates the *p*-value of FDR = 0.05. Bottom: histogram shows the distribution of the probe sets across the fold change. **d** Quantitative RT-PCR analysis of mRNA from the hippocampus of WT (*n* = 5) and *Del(3.0* *Mb)/* *+* (*n* = 5) mice. The expression of all the genes in deleted region were significantly decreased in the hippocampus of *Del(3.0* *Mb)/* *+* mice (*p* < 0.05, two-tailed Welch’s *t* test). *Actb* gene was used as a control.

Human chromosome 22q11.2 has a conserved linkage group on mouse chromosome 16 (Fig. 1a). To generate a mouse with a deletion that corresponds to the most common 3.0-Mb deletion in 22q11.2DS patients, we introduced the deletion between *Pi4ka* and *Hira* genes on mouse chromosome 16 using CRISPR/Cas9 system. We obtained a total of 95 pups from manipulated embryos and 4 (5.4%) harbored a desired deletion (Supplementary Table 4). We used founder 2 to establish a mutant line (*Del(3.0* *Mb)/* *+* , hereafter). The deletion allele of the founder was successfully transmitted through the germline (Supplementary Fig. 1a–d). The expected decrease in genomic copy number of this region was confirmed in array-based comparative genomic hybridization (array CGH) (Supplementary Fig. 1e).

### Gene expression analysis in the brain of *Del(3.0Mb)/+* mice

Gene expression microarray analysis showed that 47 probe sets in the hippocampus and 54 probe sets in the frontal cortex were differentially expressed in *Del(3.0* *Mb)/* *+* mice (false discovery rate < 0.05, above the dashed line in Fig. 1b and Supplementary Fig. 2a), and most of them were downregulated in *Del(3.0* *Mb)/* *+* mice. Quantitative RT-PCR confirmed that expression of all genes except for *Dgcr8* in the deleted region were significantly reduced to ~50% of the expression in WT mice (Fig. 1d). *Dgcr8* expression was only reduced to 73.6%, possibly due to negative feedback regulation by the Microprocessor complex containing DGCR8 itself^23^. We found that the distribution of the miRNA expression was shifted to the left (lower panels in Fig. 1c; Supplementary Fig. 2b), indicating that miRNA biogenesis was globally downregulated. Specifically, miR-185 showed the largest decrease both in the hippocampus and the frontal cortex of *Del(3.0* *Mb)/* *+* mice possibly because miR-185 is located in the deleted region. It is notable that an miR-185 target *Emc10* was upregulated in the frontal cortex of *Del(3.0* *Mb)/* *+* mice (Supplementary Fig. 2a). Furthermore, most of the other upregulated genes in the hippocampus and frontal cortex (e.g., *Lincppara* and *Spaca6*) encode primary miRNA transcripts, indicating the accumulation of substrates for the microprocessor complex.

### Cardiovascular and thymic abnormalities in *Del(3.0Mb)/+* mice

We found that neonatal mortality rate was higher in *Del(3.0* *Mb)/* *+* mice than that in WT siblings. Of 211 *Del(3.0* *Mb)/* *+* pups, 149 (70.6%) died after birth, while 77 (29.1%) out of 265 WT siblings died (Supplementary Table 5). Cardiac defects are observed in ~80% of the patients with 22q11.2DS^2,24,25^, and are a main cause of perinatal death of the patients. Therefore, we examined cardiovascular phenotype of *Del(3.0* *Mb)/* *+* embryos at embryonic day 18.5. Among 15 *Del(3.0* *Mb)/* *+* embryos, 3 (20.0%) had cardiovascular abnormalities (interrupted aortic arch, IAA; aberrant right subclavian artery, ARSA) and 7 (46.7%) showed hypoplasia of the thymus with an asymmetric appearance (Supplementary Table 6; Supplementary Fig. 3), indicating that *Del(3.0* *Mb)/* *+* mice reproduced cardiovascular and thymic abnormalities of 22q11.2DS^25–27^.

### Behavioral phenotypes of *Del(3.0Mb)/+* mice

**Fig. 2:**
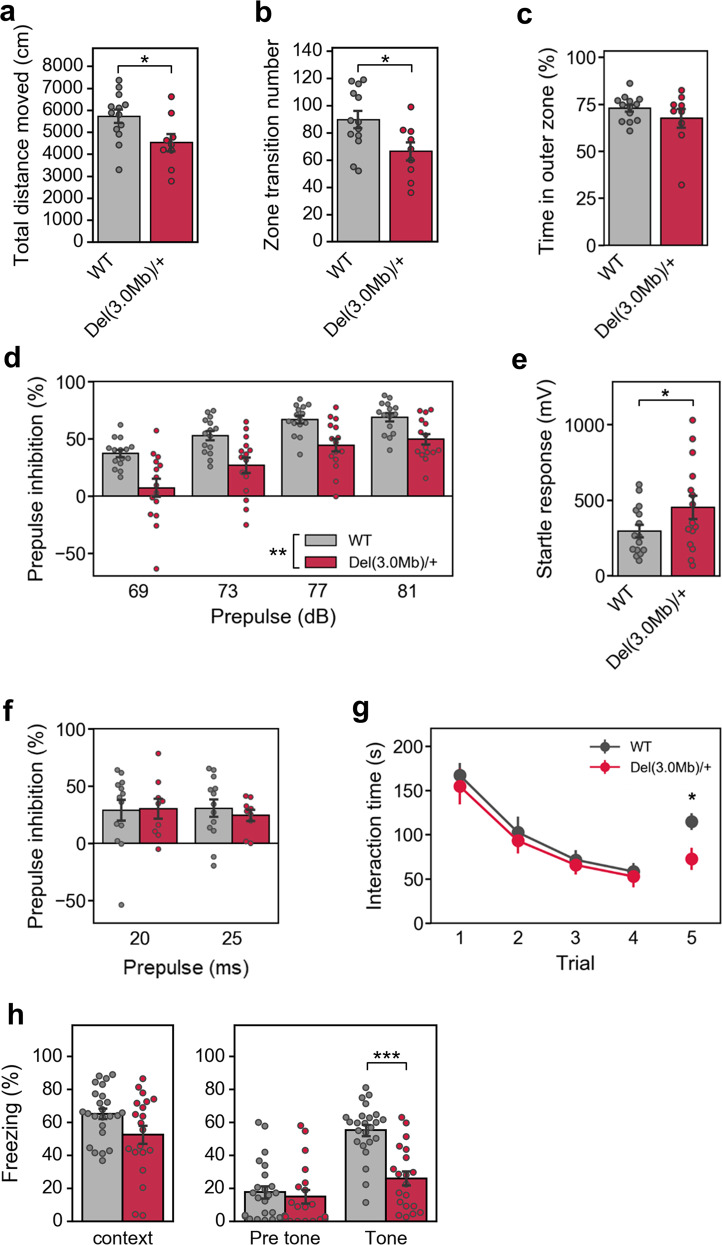
A battery of behavioral tests of *Del(3.0* *Mb)/* *+* mice. **a**–**c** Open-field test (WT, *n* = 13; *Del(3.0* *Mb)/* *+* , *n* = 9). **a** The total distance moved during the 10-min test period. **b** The number of transitions between the outer and inner zones. **c** Percentage of time spent in the outer zone. **d** Percentage of auditory prepulse inhibition (PPI) (WT, *n* = 15; *Del(3.0* *Mb)/* *+* , *n* = 15). Auditory PPI was measured at four different prepulse levels (69, 73, 77, and 81 dB). **e** Measurements of acoustic startle responses to the 120-dB startle stimulus. **f** Measurements of visual PPI (WT, *n* = 13; *Del(3.0* *Mb)/* *+* , *n* = 9). Visual PPI was measured at two conditions of light prepulse duration (20 or 25 ms). **g** Five-trial direct social interaction test (WT, *n* = 13; *Del(3.0* *Mb)/* *+* , *n* = 9). A subject mouse was habituated to the same intruder of juvenile mouse (trials 1–4) and dishabituated toward a novel mouse (trial 5). Time length spent interacting with the intruder was evaluated. Each 5-min trial was separated by a 30-min interval. **h** Fear-conditioning test. A context-dependent freezing response (%) is shown on the left panel towards the tone and foot–shock paring (conditioning) measured 24 h after the initial exposure. A cue-dependent freezing response (%) is shown on the right panel measured 48 h after the conditioning. Two-tailed Welch’s *t* tests (**a**, **b**, **c**, **e**, fifth trial of **g** and **h**) or two-way repeated-measures ANOVA (**d**, **f**, 1–4 trial of **g**). All data are expressed as mean ± SEM. *p < 0.05, **p < 0.01, ***p < 0.001.

Several studies have characterized behavioral phenotypes of the 22q11.2DS models (Supplementary Table 7), and we conducted comparable behavioral tests to the previous reports. In an open-field test, *Del(3.0* *Mb)/* *+* mice moved a shorter distance in the entire field and transited between the outer and inner zones less frequently (Fig. 2a, b). There were no differences in zone preference (Fig. 2c). As for 120-min locomotor activity in a test cage, total locomotor activity was not significantly different, but activity for the first 5 min appeared to be less in *Del(3.0* *Mb)/* *+* mice as compared with WT controls (Supplementary Fig. 4a–c). Because the previous 22q11.2DS models showed hyperactivity (Supplementary Table 7), this hypoactivity phenotype was unique to our model. We found no significant differences between WT and *Del(3.0* *Mb)/* *+* mice in rotarod and elevated-plus maze tests (Supplementary Fig. 4d–f), indicating the normal anxiety-like behavior and motor coordination and learning in *Del(3.0* *Mb)/* *+* , which is consistent with previous reports (Supplementary Table 7).

Prepulse inhibition (PPI) is one of the well-known translatable measures of sensorimotor gating; patients with schizophrenia^28–31^ and the 22q11.2DS models have shown reduced PPI (Supplementary Table 7). We evaluated PPI in accordance with a conventional protocol using auditory prepulse stimuli. As expected, it was significantly decreased in *Del(3.0* *Mb)/* *+* mice (Fig. 2d). However, the amplitude of startle response per se was significantly higher in *Del(3.0* *Mb)/* *+* mice than those of WT littermates (Fig. 2e). These results were consistent with previous reports (Supplementary Table 7).

PPI occurs when stimulation of a different modality is applied as a prepulse^32,33^. Therefore, we conducted another PPI test using light stimulation as a prepulse. Both *Del(3.0* *Mb)/* *+* and WT mice showed PPI in this paradigm and there was no difference between them (Fig. 2f), raising a possibility that reduction of the auditory prepulse-mediated PPI in *Del(3.0* *Mb)/* *+* and possibly in the other 22q11.2DS models, was caused by secondary effects of hearing impairments (see “Discussion”).

Next, we evaluated sociability and social novelty preference by the three-chamber test. There was no significant difference between WT and *Del(3.0* *Mb)/* *+* mice in the sociability and social novelty preference (Supplementary Fig. 4g, h). Because the three-chamber test does not evaluate genuine social memory, we performed a more stringent test, the five-trial direct social interaction test. In this test, a subject mouse was exposed to the same intruder mouse for four successive trials (trial 1–4). On the fifth trial, the subject mouse was exposed to a novel intruder mouse (trial 5). A previous report performing a similar test showed that *Df(16)A*^*+/−*^ mice had an impaired social memory^34,35^ (Supplementary Table 7). Unlike the previous report, there was no significant difference in trial 1–4 between groups, but *Del(3.0* *Mb)/* *+* mice exhibited significant reduction of the interaction time in trial 5, as compared with WT littermates (Fig. 2g).

A fear-conditioning test has repeatedly revealed an impairment of learning and memory in 22q11.2DS models; however, the results were still controversial (Supplementary Table 7). *Del(3.0* *Mb)/* *+* mice showed a decreased freezing time in cue-dependent fear-conditioning tests, but the freezing time in contextual fear-conditioning tests was not significantly different between two genotypes (Fig. 2h).

### Cognitive function in *Del(3.0Mb)/+* mice

As cognitive dysfunction is common in patients with schizophrenia, we next examined spatial working memory of *Del(3.0* *Mb)/* *+* mice by a Y-maze test. Although the number of mice entering to arms and alternation were smaller in *Del(3.0* *Mb)/* *+* mice as compared with WT controls (Supplementary Fig. 5a, b), there was no significant difference in spontaneous alternation, an indicator of spatial working memory (Supplementary Fig. 5c). Also, we examined a visual cognitive function by a novel object recognition test. There was no significant difference in exploration time and percentage of preference between familiar and novel objects (Supplementary Fig. 5d, e). The reported behavioral data of 22q11.2DS models in these tests are consistent with our results (Supplementary Table 7).

**Fig. 3:**
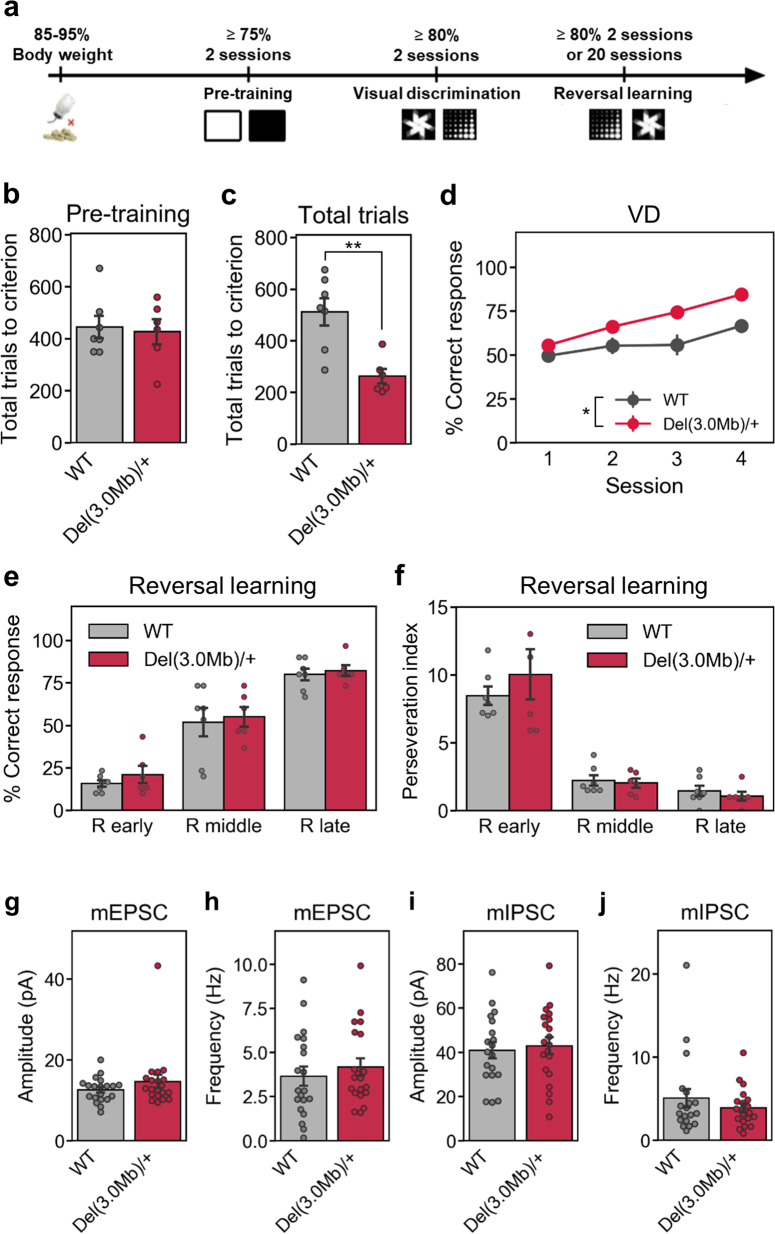
Performance of WT and *Del(3.0* Mb)/ *+* mice in visual discrimination (VD) and reversal learning. **a** Experimental schedule for visual discrimination and reversal learning tasks. **b** A total number of trials to reach the criterion in the pre-training task. **c** A total number of trials to reach the criterion in VD task. **d** Acquisition of the first four sessions in VD learning. **e** Percentage of correct responses and **f** perseveration index on the early, middle, and late stages of reversal learning. R early, first reversal learning session. R middle, session midway through reversal session. R late, final reversal session. All data are expressed as means ± SEM (WT, *n* = 7; *Del(3.0* *Mb)/* *+* , *n* = 6). *p < 0.05, **p < 0.01. **g**–**j** Basal synaptic transmission in the mPFC of *Del(3.0* *Mb)/* *+* mice. Layer 2/3 pyramidal neurons in acute mPFC slices from 2-week-old *Del(3.0* *Mb)/* *+* and WT littermates were whole-cell patch clamped and miniature excitatory postsynaptic currents (mEPSCs) and miniature inhibitory postsynaptic currents (mIPSCs) were recorded. Bar graphs show the amplitude (**g**, **i**) and the frequency (**h**, **j**) of mEPSC (**g**, **i**) and mIPSC (**h**, **j**), respectively. Data are expressed as mean ± SEM (*n* = 19–20 cells from WT control mice and *n* = 20 cells from *Del(3.0* *Mb)/* *+* mice).

To evaluate higher order cognitive functions of *Del(3.0* *Mb)/* *+* mice, we next conducted a visual discrimination (VD) learning test. In the pre-training session, mice were trained to touch a plain white square stimulus to obtain a reward (Fig. 3a). There was no significant difference in the number of trials to reach the pre-training criterion (>75% correct response for 2 consecutive days) between *Del(3.0* *Mb)/* *+* mice and WT mice (Fig. 3b). The animals were subsequently subjected to the VD task, in which mice were required to touch a stimulus to obtain a reward from a pair of visual stimuli, marble, and fan (Fig. 3a). *Del(3.0* *Mb)/* *+* mice took an approximately half of total training trials that WT mice required to reach the criterion (>80% correct response for 2 consecutive days) (Fig. 3c). The learning curve of the VD task in *Del(3.0* *Mb)/* *+* mice was significantly shifted to the leftward as compared with that in WT mice (Fig. 3d). After both groups of mice reached the learning criterion of the VD task, the reward contingencies were reversed (reversal learning task) (Fig. 3a). There were no significant differences in the correct response rate (Fig. 3e) or perseveration index (Fig. 3f) between the genotypes. In line with this, the basal synaptic transmission of *Del(3.0* *Mb)/* *+* was normal in layer 2/3 in the medial prefrontal cortex (mPFC), a region that is thought to be important for reversal learning (Fig. 3g–j).

### Visual-evoked potentials (VEP) in *Del(3.0Mb)/+* mice

**Fig. 4:**
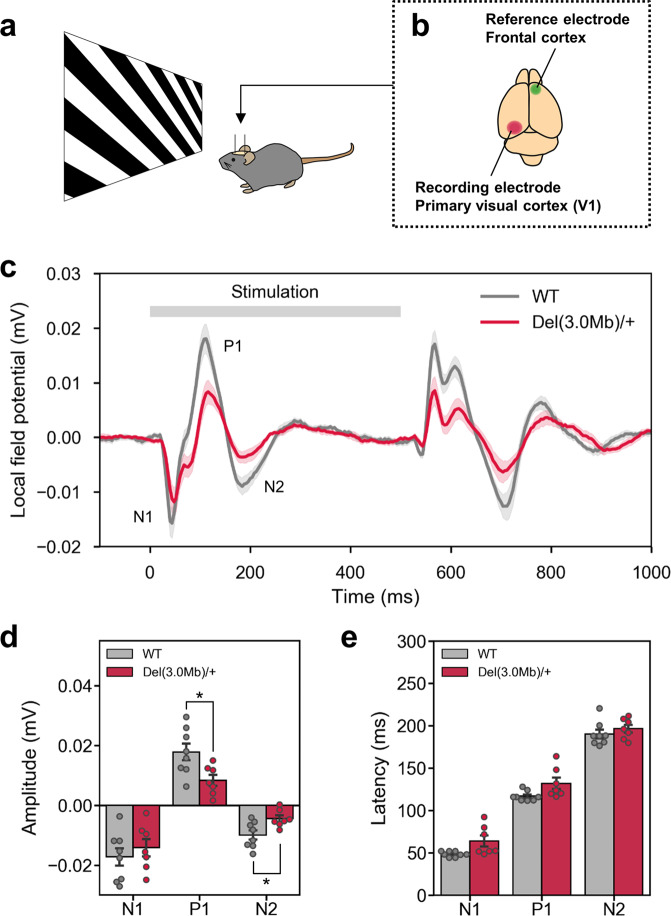
Measurements of the visual-evoked potential in *Del(3.0* *Mb)/* *+* mice. **a** Schematic drawing of the experimental setup for measuring the visual-evoked potential. **b** A recording electrode and a reference electrode were implanted into the primary visual cortex (V1) and the frontal cortex, respectively. **c** The wave forms of the grand mean of time-averaged local field potential. **d** The mean amplitudes of negative and positive potentials (N1, P1, and N2 indicated in **c**). **e** The mean latency of negative and positive potential peaks (N1, P1, and N2). All data are expressed as means ± SEM (WT, *n* = 8; *Del(3.0* *Mb)/* *+* , *n* = 7). *p < 0.05.

Because impairment in early visual sensory processing has been reported in individuals with schizophrenia and their first relatives, we investigated visual-evoked potentials (VEP) of *Del(3.0* *Mb)/* *+* mice. We recorded local field potentials (LFP) from the V1 area of awake mice, while they are exposed to visual stimuli of square-wave gratings (Fig. 4a, b). VEP responses occurred at the start (ON response) and the end of the stimulus (OFF response) (Fig. 4c). In the ON response, peaks of the N1, P1, and N2 components appeared at ~50 ms, ~120 ms, and ~190 ms from the onset of the stimulus, respectively (Fig. 4c). The polarity and latency of the components were similar to those observed in humans^36^. We observed generally smaller VEP in *Del(3.0* *Mb)/* *+* than in WT littermates (Fig. 4c). The amplitudes of P1 and N2 peaks were significantly smaller in *Del(3.0* *Mb)/* *+* mice (Fig. 4d). The latency of each peak (N1, P1, and N2) was not significantly different between two genotypes (Fig. 4e).

### Circadian behavioral rhythm in *Del(3.0Mb)/+* mice

**Fig. 5:**
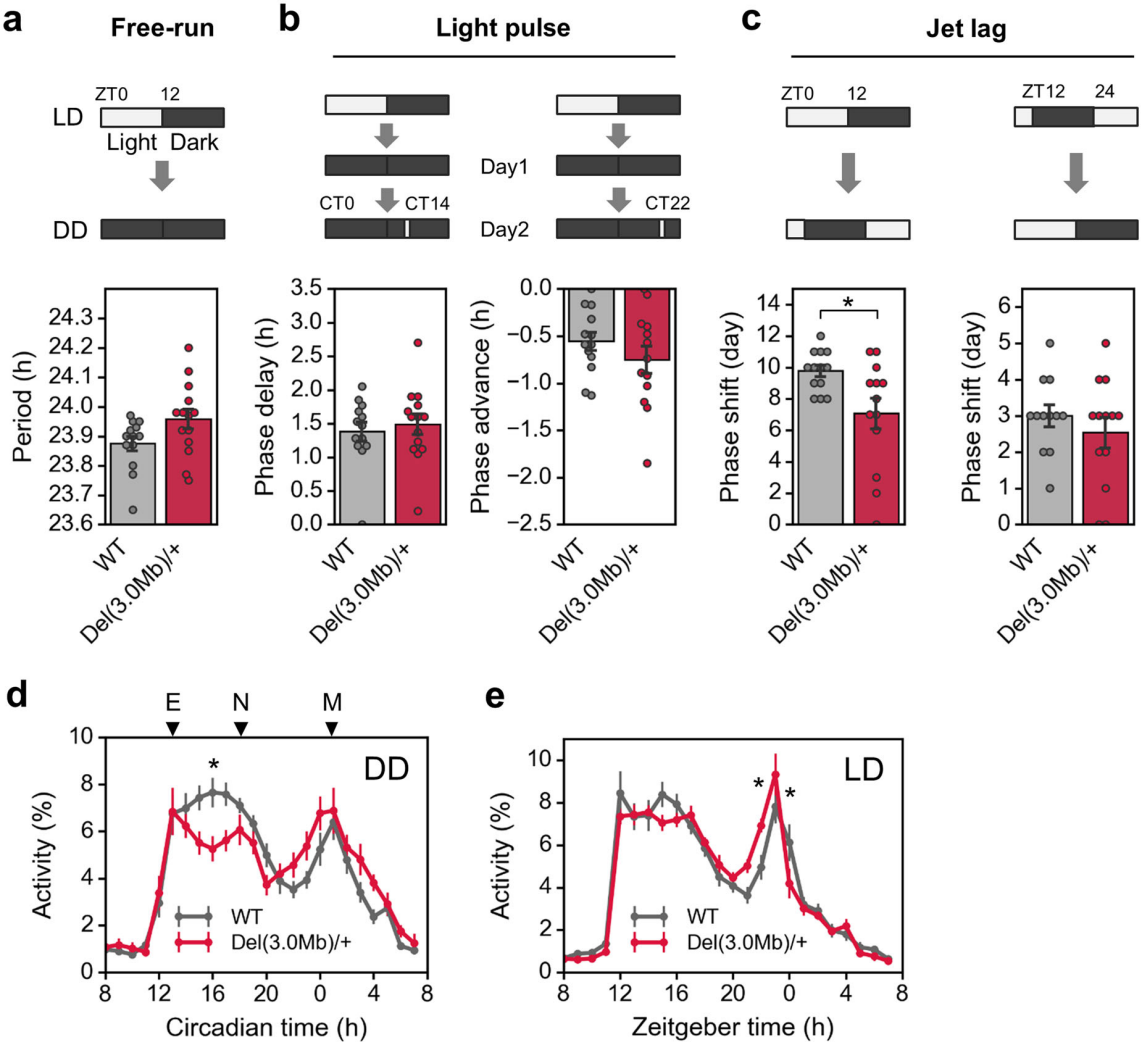
Circadian activity and its re-entrainment by ambient light. **a** The upper panel shows a schematic diagram of light–dark cycle. Spontaneous locomotor activities of WT mice (*n* = 13) and *Del(3.0* *Mb)/* *+* (*n* = 14) were measured by an infrared thermal sensor. Free-running period of circadian behavioral rhythm was calculated by a χ^2^-periodogram (lower panel). **b** Phase shifting responses to a 30-min light pulse at CT14 (WT, *n* = 13; *Del(3.0* *Mb)/* *+* , *n* = 14) and at CT22 (WT, *n* = 13; *Del(3.0* *Mb)/* *+* , *n* = 13). **c** Responses to rescheduled light–dark cycles with 8-h advance (WT, *n* = 13; *Del(3.0* *Mb)/* *+* , *n* = 13) and 8-h delay (WT, *n* = 12; *Del(3.0* *Mb)/* *+* , *n* = 13). The number of days required to adapt to a new light–dark cycle was counted. **d**, **e** Activity profile in DD (**d**) and LD (**e**) (WT, *n* = 13; *Del(3.0* *Mb)/* *+* , *n* = 14). Data from day 7 through 17 in constant dark condition were used for the calculation of the circadian periods and the activity profiles. *p < 0.05 (Sidak’s multiple comparison test).

Sleep problems are common in patients with 22q11.2DS and psychiatric disorders^37–40^, and disturbances in circadian rhythm have been implicated in various psychiatric disorders^41^. Therefore, we finally investigated circadian rhythm of our 22q11.2DS model mice (Fig. 5a). A χ^2^ periodogram analysis of their spontaneous locomotor rhythm in a constant dark condition (DD) revealed that the free-running period was unaltered in *Del(3.0* *Mb)/* *+* mice (Fig. 5a). The vulnerability of sleep–wake cycle to the ambient light condition was assessed by light-pulse and jet lag tests. In the light-pulse test, animals were first entrained to 12 h: 12 h light–dark (LD) cycles for at least 2 weeks and then reared in DD for 1 day, and in the next day, they were exposed to a 30-min light pulse in the early or late subjective night (CT14 or CT22; CT, circadian time). The resulting phase shift of *Del(3.0* *Mb)/* *+* mice after the light exposure was comparable with that of WT controls (Fig. 5b). In the jet lag test, animals were entrained to the LD cycles for at least 2 weeks, and then the phase of the light–dark cycle was advanced or delayed by 8 h. *Del(3.0* *Mb)/* *+* mice adapted to a new light–dark cycle faster than WT controls when the phase was advanced (Fig. 5c). These results demonstrated that *Del(3.0* *Mb)/* *+* mice were more sensitive to an environmental light condition than WT controls, or alternatively that the robustness of the circadian clockwork is reduced in the mutant.

Furthermore, we found that activity levels were significantly reduced in *Del(3.0* *Mb)/* *+* mice at around CT16 in DD (Fig. 5d), resulting in an emergence of a third peak (previously termed as a night peak, N; see ref. ^42^) between the evening (E) and morning (M) peaks (Fig. 5d). In LD, on the other hand, the night peak was not as obvious as in DD, and temporal activity profile was similar between *Del(3.0* *Mb)/* *+* and WT mice (Fig. 5e). These observations indicate that an intrinsic circadian rhythm was disturbed in *Del(3.0* *Mb)/* *+* , but light-induced entrainment can adjust the disturbed behavioral rhythm in the mutant mice.

## Discussion

In this study, we have generated a novel 22q11.2DS mouse model, *Del(3.0* *Mb)* *+* mice, by using CRISPR/Cas9 system in C57BL/6N genetic background. Our model has significant advantages, because *Del(3.0* *Mb)/* *+* mice reproduced the 3.0-Mb deletion that ~90% of patients have, while the other existing models only mimic a minor 1.5-Mb deletion^16–19,43^. We have not investigated whether CRISPR/Cas9-mediated off-target mutations were introduced in the untargeted genomic loci. Therefore, there is a possibility that these phenotypes are influenced by unintended mutations, although potential off-target sites for sgRNAs in this study are located in the intergenic regions or introns.

It has been reported that the 1.5-Mb deleted region contains key genes responsible for the increased risk of mental disorders. In fact, some genes within the 1.5-Mb region (e.g., *Tbx1*, *Dgcr8*, *Prodh*, and *Sept5*) have been identified as potentially associated with phenotypes relevant to psychiatric disorders by animal model studies^44^. On the other hand, the role of the genes existing in the region deleted only in our model (1.4-Mb region) in psychiatric symptoms of 22q11.2DS is unclear.

A characteristic difference between *Del(3.0* *Mb)/* *+* mice and the other 22q11.2DS models is hypoactive: while *Df(16)A*^*+/−*^ and *Lgdel/* *+* mice were more active than WT controls in the open-field test^18,35,45^, *Del(3.0* *Mb)/* *+* mice traveled shorter distance. Also, *Del(3.0* *Mb)/* *+* mice entered arms less frequently in the Y-maze test, and circadian rhythm analysis revealed that *Del(3.0* *Mb)/* *+* mice showed lower activity in their subjective night. This hypoactive phenotype of *Del(3.0* *Mb)/* *+* model may reproduce instant fatigability, a frequent complaint among patients with 22q11.2DS^46^, or the negative symptoms of schizophrenia. Our results suggest that the 1.4-Mb region contains genes causing the hypoactivity of the disease.

The impairment of basal social interaction or facial recognition has been observed in individuals with 22q11.2DS^47–52^. In our genuine social interaction test, *Del(3.0* *Mb)/* *+* mice showed deficit in social recognition of a novel mouse. This phenotype of *Del(3.0* *Mb)/* *+* mice probably reproduces the impairment of social recognition observed in 22q11.2DS patients. Meanwhile, social memory of *Del(3.0* *Mb)/* *+* mice was intact (Fig. 2g). These results are inconsistent with the unimpaired sociability and social memory impairment of *Df(16)A*^*+/−*^ mice shown in previous studies, which conducted direct interaction test^34,35^ (Supplementary Table 7). The contradiction in social behavior between *Del(3.0* *Mb)/* *+* and *Df(16)A*^*+/−*^ mice may arise from the difference of the genetic background (Supplementary Table 7). Our mice are pure C57BL/6N genetic background, while *Df(16)A*^*+/−*^ mice were hybrids between 129S7/SvEvBrd-*Hprt*^*b-m2*^ and C57BL/6J.

Reduction in PPI is a well-known endophenotype of schizophrenia and has been consistently reported in other 22q11.2DS mouse models (Supplementary Table 7). In this study, a reduction in PPI was observed when auditory prepulse stimulations were used, but PPI was preserved when light was presented as prepulse. This result argues against the conserved sensorimotor deficit in 22q11.2DS models. One possible explanation of the discrepancy between the prepulse modalities is that *Del(3.0* *Mb)/* *+* mice might have a hearing abnormality. Chronic otitis media is a frequent complaint among 22q11.2DS patients^53^ and was suggested to cause a hearing difficulty in *Df1/* *+* mice^54^. Therefore, the reduction in auditory prepulse-mediated PPI in *Del(3.0* *Mb)/* *+* mice might be caused by a hearing deficit of tiny prepulse sounds rather than sensorimotor gating impairment, as suggested in a previous report^54^. Furthermore, it has recently been reported that an increase in acoustic startle response (ASR) correlates with PPI reduction in human and mouse^55–57^. Thus, the PPI reduction might be due to the increased ASR in *Del(3.0* *Mb)/* *+* mice, making it difficult to interpret the result of PPI analysis. Altogether, *Del(3.0* *Mb)/* *+* mice apparently reproduced the sensorimotor deficit of schizophrenia patients, but further analyses are required to reveal what caused the PPI reduction in our model.

In the Y-maze test and the VD test, we observed no apparent cognitive deficits in *Del(3.0* *Mb)/* *+* mice. *Del(3.0* *Mb)/* *+* mice learned the VD task faster than WT mice. This result is consistent with the previous report using a different mouse model of the 22q11.2DS^19^. Functional compensatory changes may protect mice suffering from main neural molecular effects and profound cognitive impairments^19^. Alternatively, 22q11.2 may encode some molecules that limit VD learning. In contrast, there was no difference in performance of reversal learning task between two groups of mice, suggesting that the behavioral flexibility is intact in *Del(3.0* *Mb)/* *+* mice. Taken together, these results suggest that 22q11.2 microdeletion slightly enhances ability of VD learning, but not flexibility in mice. Our electrophysiological data from layer 2/3 pyramidal neurons in the medial prefrontal cortex of 2-week-old *Del(3.0* *Mb)/* *+* mice suggest that basic parameters of excitatory and inhibitory synaptic transmission are not affected (Fig. 3g–i). Short-term depression (STD) was enhanced, whereas short-term potentiation (STP) and long-term potentiation (LTP) were both reduced in *Df(16)A*^*+/−*^ mice^34,35^. Thus, an interesting future study would be to examine synaptic transmission from layer 2 to layer 5, and also to test STD, STP, and LTP at this synapse in our *Del(3.0* *Mb)/* *+* mice to further investigate possible correlation between changes in synaptic function and abnormal behaviors observed in *Del(3.0* *Mb)/* *+* mice.

*Del(3.0* *Mb)/* *+* mice displayed a smaller VEP to the static grating stimulations with a significant decrease in the amplitude of the P1 and N2 components, but not of N1. Because the N1 component has been shown to reflect the input from the lateral geniculate nucleus to the layer 4 in the mouse primary visual cortex^58^, our results indicate that the visual thalamocortical circuits were relatively intact in *Del(3.0* *Mb)/* *+* mice, which contrasts to the deficit of auditory thalamocortical projection in another 22q11.2DS model^59^. Meanwhile, the reduced P1 and N2 amplitude suggest impaired interlaminar connectivity. The reduced amplitude of VEP, especially that of the P1 component has been recurrently reported in patients with schizophrenia^60–66^ as well as their clinically unaffected relatives, suggesting this deficit as a genetic marker for this disorder^67^. Therefore, the reduced VEP of *Del(3.0* *Mb)/* *+* mice strongly suggested its validity as a model of schizophrenia. The deficits in early visual processing of schizophrenia have also been demonstrated by apparent dysfunction in visual sensory-perceptual tasks in patients^68–73^. The faster acquisition of the VD task described above might reflect alterations in visual perceptual processing in *Del(3.0* *Mb)/* *+* mice.

Interestingly, this study revealed an impairment of rhythmic activity as a 22q11.2DS model for the first time. *Del(3.0* *Mb)/* *+* mice adapted faster to the experimental jet lag with 8-h advancement. This was consistent with the result of the light-pulse test, where the phase advance tended to be greater in *Del(3.0* *Mb)/* *+* mice. These results suggest that the circadian rhythm of *Del(3.0* *Mb)/* *+* mice is more sensitive to an environmental light condition. It is currently unknown how the fast-circadian clock resetting was achieved in *Del(3.0* *Mb)/* *+* mice, but the impairment of miRNA biogenesis might be a candidate mechanism. A number of miRNAs have been identified to show rhythmic expression under control of the circadian clock, and moreover, they can regulate output of the circadian clock via posttranscriptional regulation of the core molecular clock genes in the suprachiasmatic nucleus (SCN) and in peripheral tissues (for review, see ref. ^74^). The most striking example is miR-132, which has been identified as a negative regulator of photic entrainment of circadian rhythm; that is, knocking-down of the miRNA in the SCN potentiated the light-induced clock resetting^75^. Although the expression of miR-132 was not significantly changed in *Del(3.0* *Mb)/* *+* cortex and hippocampus, compromised biogenesis of miR-132 as well as other miRNA molecules might occur in other brain regions especially in the SCN, potentially leading to the defect in entrainment of circadian rhythm in *Del(3.0* *Mb)/* *+* mice.

Furthermore, *Del(3.0* *Mb)/* *+* mice showed a characteristic temporal activity profile with an additional peak (termed night peak) at around CT16 in DD. Previously, we found a similar daily activity profile in CaMKIIαK42R mice, which expressed a kinase-dead mutant of CaMKIIα^42^. This is particularly interesting because abnormalities in CaMKII have been found in several psychiatric disorders, including schizophrenia. Our results opened a new avenue for deciphering the relationship between circadian disruption and psychiatric disorders in 22q11.2DS.

In this study, we established a novel animal model of 22q11.2DS with an equivalent deletion to the human 3.0-Mb deletion at the 22q11.2 locus. Our model shows a series of phenotypes that reflect the symptoms observed in patients with 22q11.2DS and schizophrenia. Hence, it is a useful resource to study pathophysiology of schizophrenia associated with 22q11.2DS.

## Supplementary information

Spplemental Information
