# Supplementary material for: Comprehensive analysis of a novel mouse model of the 22q11.2 deletion syndrome: a model with the most common 3.0-Mb deletion at the human 22q11.2 locus
Source: Transl Psychiatry. 2020 Feb 5;10:35. doi: 10.1038/s41398-020-0723-z (PMC7026107; doi:10.1038/s41398-020-0723-z)
Supplement: Supplementary file 1 — Spplemental Information [file 41398_2020_723_MOESM1_ESM.docx]

**Comprehensive analysis of a novel mouse model of the 22q11.2 deletion syndrome: A model with the most common 3.0-Mb deletion at the human 22q11.2 locus**

***Supplemental Information***

**Supplemental Methods**

**Preparation of *Cas9* mRNA, sgRNAs and a single-stranded oligodeoxyribonucleotide (ssODN)**

We used CRISPRdirect software to design sgRNA with smaller number of off-target sites, (<http://crispr.dbcls.jp>; (1)). Two pairs of sgRNAs were positioned on either endpoint of the deletion. The protospacer sequences of sgRNAs were listed in Supplemental Table 1. We constructed pDR274 plasmids (Addgene #42250) encoding each of the sgRNA. The sgRNAs were transcribed *in vitro* using the *Dra*I-digested pDR274 vectors as a template and the MEGAshortscript T7 kit (Ambion, CA, USA) according to manufacturer’s instruction. The synthesized sgRNAs were then purified using MEGAclear kit (Ambion) according to manufacturer’s instruction. The *Cas9* mRNA was transcribed *in vitro* using an *Age*I-digested Cas9 expression vector, pcDNA3.1-hCas9 (2), as a template and the MessageMax T7 ARCA-Capped Message mRNA transcription kit (Cellscript, WI, USA) according to the manufacturer’s instruction. The poly(A) tailing reaction was performed using A-plus Poly(A) Polymerase Tailing kit (Cellscript) according to the manufacturer’s instruction. *Cas9* mRNA was then purified using MEGAclear kit (Ambion) according to the manufacturer’s instruction. The ssODN designed to bridge the deletion endpoints were 120 nucleotides in length and positioned directly adjacent to the most external sgRNA site.

**Microinjection of *hCas9* mRNA, sgRNAs and ssODN**

Fifty ng/μL of hCas9 mRNA, 25 ng/μL of sgRNA (each) and 100 ng/μL of ssODN were mixed in RNase-free water and microinjected into the cytoplasm of C57BL/6N (Charles River Laboratories Japan Inc., Kanagawa, Japan) fertilized eggs. Survived microinjected embryos were cultured in modified Whitten’s medium (mWM) until the 2-cell stage. Injected embryos were transferred into oviducts of 0.5-day-post-coitum recipients (ICR, Charles River Laboratories Japan Inc.). Obtained founder candidate mice were genotyped and crossed with C57BL/6N mice to obtain N1 mice.

**Genotyping of *Del(3.0Mb)/+* mice by PCR assay**

The screening of mutant mice was performed by PCR assay using Ex *Taq* DNA polymerase (Takara Bio Inc., Shiga, Japan). Genomic DNA was extracted from placenta or tail tips of mice or embryos. The following primers were used for genotyping of the mice and embryos: Del3.0Mb-Fw, 5’-CTTGCATATTTCACGGAGGCG-3’; Del3.0Mb-Rv, 5’-CAAGTAGAGAGGGAGTGGTGC-3’. The PCR condition was 98°C for 2 min, 30 cycles of melting at 98°C for 30 s, annealing at 65°C for 30 s, and extension at 72°C for 30 s, with additional extension at 72°C for 2 min at the end. PCR products were analyzed in 1% agarose gel electrophoresis and the sequences were confirmed by DNA sequencing (Fasmac Co., Kanagawa, Japan).

**Array comparative genomic hybridization (array CGH) analysis**

To confirm a copy number loss (or deletion) on chromosome 16qA13, we conducted array CGH analysis according to previously reported methods (3). Array CGH was performed using an Agilent SurePrint G3 Mouse CGH 4x180K Microarray (Agilent, Santa Clara, CA, USA) according to the manufacturer’s instructions. CNV calls were made with Nexus Copy Number software v9.0 (BioDiscovery, El Segundo, CA, USA) using the Fast Adaptive States Segmentation Technique 2 (FASST2) algorithm, which is a hidden Markov model-based approach. The log_2_ ratio threshold for copy number loss (or deletion) was set at −0.4. The significance threshold to adjust the sensitivity of the segmentation algorithm was set at 1 × 10^−6^, and at least five contiguous probes were required for CNV calls. Genomic locations are reported in NCBI Build 37/UCSC mm9 coordinates.

**mRNA microarray analysis**

Preparation of cRNA, hybridization, and scanning of microarrays were performed according to the manufacturer’s protocol. Briefly, biotinylated cRNAs were synthesized by GeneChip 3’ IVT PLUS Reagent Kit (Affymetrix, CA, USA) from 250 ng total RNA. Biotinylated cRNA yields were checked with the NanoDrop ND-2000 Spectrophotometer. Following fragmentation, 15 μg of cRNA was hybridized for 16 h at 45°C on GeneChip Mouse Genome 430 2.0 Array. Arrays were then washed and stained in the GeneChip Fluidics Station 450 (Affymetrix), followed by scanning in an Affymetrix 3000 7G scanner. Background subtraction, normalization and summarization were performed using the robust multi array average algorithm (RMA) method by Expression Console Software (Affymetrix). We analyzed 3 *Del(3.0Mb)/+* mice and 3 WT mice. An empirical Bayes moderated *t*-statistics was calculated by using limma eBayes package. Benjamini and Hochberg’s FDR was used to control false positives caused by multiple testings.

**miRNA microarray analysis**

Preparation of labeled miRNA, hybridization, and scanning of microarrays were performed according to the manufacturer’s protocol. Cyanine-3 (Cy3) labeled miRNA was prepared from 100 ng total RNA using the miRNA Complete Labeling and Hyb Kit (Agilent). The dried Cy3-labeled miRNA was resuspended in 18 μL of nuclease-free water, then 4.5 μL of the 10xGE Blocking Agent and 22.5 μL of 2× HiRPM Hybridization Buffer were added. After incubation at 100°C for 5 min, the samples were immediately transferred to an ice water bath and hybridized to Mouse miRNA V21.0 Microarray (Agilent) at 55°C for 20 h in a rotating Agilent hybridization oven. After hybridization, microarrays were washed with GE Wash Buffer 1 (Agilent) at room temperature for 5 min and with GE Wash Buffer 2 (Agilent) at 37°C for 5 min, then dried immediately. Microarrays were scanned immediately after washing on the Agilent SureScan Microarray Scanner (G2600D) using one color scan setting for 8x15k array slides (Scan Area 61 × 21.6 mm, Scan resolution 5 μm, Dye channel is set to Green PMT is set to XDR Hi 100% and XDR Lo 5%). The scanned images were analyzed with Feature Extraction Software 12.0.3.1 (Agilent) using default parameters to obtain background subtracted and spatially detrended Processed Signal intensities. The GeneView files were generated using Agilent Feature Extraction software version 12.0.3.1. We analyzed 3 *Del(3.0Mb)/+* mice and 3 WT mice. For statistical analysis, we used a set of miRNAs that were detected in all the 6 samples (428/1881 (22.8%) in the hippocampus; 484/1881 (25.7%) in the frontal cortex).

**Quantitative RT-PCR**

For quantification of mRNA, first strand cDNA was synthesized using PrimeScript RT reagent Kit (Perfect Real Time) (Takara Bio) according to the manufacturer’s instruction. Briefly, 300 ng of total RNA was reverse transcribed using 25 pmol of oligo dT primer and 50 pmol of random 6 mer in 10 μL reaction. The resultant cDNA was diluted at 1:30 ratio in TE. For quantification of miRNA, cDNA was synthesized using Mir-X miRNA First-Strand Synthesis Kit (Takara Bio) according to the manufacturer’s instruction. Briefly, ~1 μg of total RNA was reverse transcribed in 10 μL reaction. The resultant cDNA was diluted at 1:10 ratio in nuclease-free water. All samples within an experiment were reverse transcribed at the same time. All real-time PCR reactions were performed using the PowerUp SYBR Green Master Mix (Applied Biosystems, CA, USA) and the StepOnePlus Real-Time PCR system (Applied Biosystems). The experiments were carried out in triplicate for each data point. The relative quantification in gene expression was determined using ∆∆Ct method (4). The sequences of the primers are listed in Supplemental Table 2.

**Histology**

In order to obtain the embryos, we conducted *in vitro* fertilization between C57BL/6N WT eggs and N1 *Del(3.0Mb)/+* sperm. Subsequently, viable 2-cell embryos were transferred into oviducts of pseudopregnancy mice (ICR). Embryos (N2) were isolated from 18.5-day pregnant female mice by the Cesarean section, where the day of vaginal plug was scored as embryonic day (E) 0.5. Samples were subjected to a thoracic incision and fixed with 4% paraformaldehyde (PFA) in 0.1 M phosphate buffer (PB), at 4°C overnight. Then, organs were dissected and observed using stereoscopic microscope LEICA MZ6 (Leica, Germany) and camera IC90E (Leica).

**Open field test**

Mice were placed in the center of the open field (diameter: 75 cm, height: 35 cm) and were allowed to explore freely the arena for the following 10 min under moderately light conditions (15 lux). Their movement was recorded with a camera mounted above the arena, and their activity was measured automatically using smart 3.0 software (Bio Research Center Co. Ltd., Nagoya, Japan). The open field was divided into an inner circle (diameter: 50 cm), and an outer area surrounding the inner circle. Measurements included total distance moved and time spent in the inner and outer sections. The open field arena was cleaned with 70% ethanol and wiped with paper towels between each trial.

**Y-maze test**

The Y-maze apparatus consists of three equal-sized plywood arms (50 cm long, 12 cm high, and 4 cm wide). Each arm of the Y-maze was positioned at an equal angle. Each mouse was placed at the cross points of arms and allowed to move freely through the maze for an 8-min session under moderately light conditions (35 lux). The movement of mice was recorded with a camera mounted above the apparatus. The number of arm entries was counted manually. Spontaneous alternation behavior was defined as the entry into all three arms (i.e., arm A, arm B, and arm C) on consecutive choices in triplet set (i.e., ABC, ACB, BAC, BCA, CAB, and CBA). The percentage of spontaneous alternation behavior was calculated as the ratio of actual to possible alternations (defined as the total number of arm entries − 2) × 100 as described previously (5). The Y-maze apparatus was cleaned with 70% ethanol and wiped with paper towels between each trial.

**Elevated plus-maze test**

The elevated plus-maze consists of two opposing open (25 cm × 8 cm) and two closed arms (25 cm × 8 cm × 20 cm), linked by a neutral area (8 cm × 8 cm) in the center of the apparatus (6). The entire apparatus was elevated to height of 50 cm above floor level. The light intensity around the maze was set at 100 lux. Mice were placed in the central platform facing an open arm and allowed to move freely through the maze for 5 min. Their behavior was recorded with a camera mounted above the apparatus. Measurements included the number of entries to and closed arms entries and time spent open and closed arms. The maze was cleaned with 70% ethanol and wiped with paper towels between each trial.

**Locomotor activity under the novel environment**

The locomotor activity test was performed according to previously reported methods (7). Each individual mouse was placed in a standard transparent rectangular rodent cage (25 cm × 30 cm × 18 cm) under moderately light conditions (15 lux). The movement of mice was detected for 120 min using a pyroelectric infrared sensor (NS-AS01; Neuroscience, Tokyo, Japan) placed over the cage and locomotor activity was measured using Digital acquisition system (NS-DAS-8; Neuroscience).

**Novel object recognition test**

The novel object recognition test was carried out as described previously (8). The arena used for the test was an open box (30 cm × 30 cm × 35 cm) covered with fresh bedding. The test procedure consists of three sessions: habituation, training, and retention. During the habituation session, mice were individually habituated to the open box, in the absence of objects with 10 min of free exploration period for 3 consecutive days. In the training session, two novel objects of similar size, but different shape and color, were placed in the arena and the animals were allowed to explore for 10 min under moderately light conditions (15 lux). Time spent exploring each object was recorded. In the retention session, the animals were placed back into the same box 24 h after the training session, one of the familiar objects used during training was replaced by a novel object, and the mice were allowed to explore freely for 5 min. The preference index in the retention session, the ratio of the amount of time spent exploring the novel object over the total time spent exploring both objects, was used to measure cognitive function. In the training session, the preference index was calculated as the ratio of time spent exploring the replaced novel objects to the total exploration time.

**Five-trial direct social interaction test**

Subject mice were placed individually into home cage (45 cm × 28 cm × 16 cm) for 1 h before starting test under the moderately light conditions (15 lux). A juvenile intruder mouse (5-week- old) was introduced into the subject mouse’s home cage. The subject mouse was exposed to the same intruder mouse for 5 min over 4 trials with an inter-trial-interval of 30 min. During the fifth trial, the subject was exposed to a novel intruder mouse (5-week-old). The time spent in social interaction (close following, inspection, anogenital sniffing, and other social body contacts) was recorded.

**Three-chamber sociability and social novelty tests**

The sociability and social novelty tests (9) apparatus is a rectangular, three-chambered opaque Plexiglas boxes (each box is 20 cm long, 40.5 cm wide, 22 cm high). Dividing walls were made from clear Plexiglas, with small square openings allowing access into each chamber. A small cylindrical cage made of Plexiglas with multiple holes (diameter: 7 cm, height: 12 cm) was used as the cage enclosing a stranger mouse, which allowed nose contact through the holes, but prevented fighting. A weight (cup) was placed on the top of the cage to prevent the test mouse from climbing. Two cylindrical cages were located in the left and right chambers during the trial. The chambers of the social apparatus and two cylindrical cages were cleaned with 70% ethanol and wiped with paper towels between each trial. Before testing day, mice were individually habituated to the apparatus, with 10 min of free exploration period for 3 consecutive days. On the testing day, the test mouse was placed in the middle chamber and allowed to explore freely for 10 min (habituation phase). After habituation phase, an unfamiliar C57BL/6J mouse with the same age and sex (stranger1) that had no prior contact with the subject mice, was placed in one of the side boxes. The cylindrical cage on the other side remained empty. The location of stranger 1, the left versus right side chamber was systematically alternated between trials. The test mouse was placed in the middle chamber and allowed to explore freely the entire test box for 10 min. Sniffing zone was defined as the range of 10 cm in radius from each center point of cylindrical cages. Time spent in each box and in each sniffing zone, and the number of entries into each box were recorded using Ethovision automated tracking program (Brainscience Idea Co., Ltd., Osaka, Japan) to measure the sociability. After the end of the first 10 min, each mouse was subjected to the a second 10-min session to measure social preference for a new stranger. In the second session, unfamiliar mouse was placed in the box that had been empty during the first 10-min session. This second stranger (stranger 2) was also enclosed in an identical small cylindrical cage. The test mouse had a choice between the stranger 1 and stranger 2. As described above, time spent in each box and in each sniffing zone, and the numbers of entries into each box were recorded to measure the preference for social novelty.

**Auditory prepulse inhibition**

The auditory prepulse inhibition (PPI) test was performed by using SR-Lab system (San Diego Instruments, San Diego, CA, USA), as described previously (10, 11). A standard startle chamber applicable to mice and rats (San Diego Instruments) was used. The startle chamber consisted of a Plexiglas cylinder for mice (105 mm, 38 mm inner diameter, 50 mm outer diameter) and was placed in a sound-attenuated chamber, in which the animals were individually placed. The cylinder was mounted on a plastic frame under which a piezoelectric accelerometer was placed to record and transduce the vibrations of the cylinder. After the animals were placed in the chamber under moderately bright light conditions (180 lux), they were allowed to acclimate for 10 min, during which they are exposed to 65 dB background white noise was continually present. Individual mouse then received 10 startle trials, 10 no-stimulus trials and 40 PPI trials. The inter-trial interval was between 10 and 20 s and each session lasted 17 min. The startle trial consisted of a single 120 dB white noise burst lasting 40 ms. PPI trials consisted of a prepulse (20 ms burst of white noise at 69, 73, 77 or 81-dB intensity) followed by the startle stimulus (120 dB, 40 ms white noise) 100 ms later. Each of the four prepulse trials (69, 73, 77 or 81 dB) was done 10 times. Sixty different trials were performed pseudorandomly to ensure that each trial was done 10 times and that no two consecutive trials were identical. The movement of the animal in the startle chamber was measured for 100 ms after the onset of startle stimulus onset (sampling frequency 1 kHz), rectified, amplified and processed by a computer, which calculated the maximal response over the 100-ms period. Basal startle amplitude was determined as the mean amplitude of the 10 startle trials. PPI (%) was calculated as follows: 100 × (pulse-alone response − prepulse-pulse response)/pulse-alone response, in which prepulse-pulse response was the mean of the 10 PPI trials (69, 73, 77 or 81 dB) and pulse-alone response was the basal startle amplitude.

**Visual prepulse inhibition**

The visual prepulse inhibition (PPI) test was conducted as previously described (12) with minor modifications. The visual prepulse was created by turning on nine light-emitting diode (LED) light bulbs in startle chamber. The light instrument was located 8 cm above the Plexiglas cylinder and the light condition of the chamber was dim (2 lux). In this test, two conditions of light prepulse duration (20 or 25 ms) were tested. The light intensity was fixed at 900 lux. After the animals were placed in the chamber, they were allowed to acclimate for 10 min, during which they are exposed to 65 dB background white noise was continually present. Individual mouse then received 10 startle trials, 10 no-stimulus trials and 20 PPI trials. The inter-trial interval was between 10 and 20 s. The startle trial consisted of a single 120 dB white noise burst lasting 40 ms. PPI trials consisted of a light prepulse (900 lux, 20 or 25 ms duration) followed by the startle stimulus (120 dB, 40 ms white noise) 100 ms later. Each of the two prepulse trials (20 or 25 ms) was done 10 times. Forty different trials were presented pseudorandomly, to ensure that each trial was done 10 times and that no two consecutive trials were identical. The movement of the animal in the startle chamber was measured for 100 ms after the onset of startle stimulus onset (sampling frequency 1 kHz), rectified, amplified and processed by a computer, which calculated the maximal response over the 100-ms period. Basal startle amplitude was determined as the mean amplitude of the 10 startle trials. PPI (%) was calculated as follows: 100 × (pulse-alone response − prepulse-pulse response)/pulse-alone response, in which prepulse-pulse response was the mean of the 10 PPI trials (20 or 25 ms) and pulse-alone response was the basal startle amplitude.

**Rota-rod test**

We performed the rotarod test using Rota-rod treadmill for mice MK-600 (Muromachi Kikai Co., Ltd., Japan). The apparatus consists of a set of five horizontal rods. These rods are separated by opaque disks. Hence, test mice cannot be distracted from one another. Mouse was placed on a still rod for 1 min. Then, the rotation was started and the time length before fall was recorded. The speed of the rod’s rotation was 6 rpm in the training session of 3 consecutive days (day1–3) and 12 rpm in the test session (day4). Six trials per day were carried out, and a maximum time length for each training was 2 min. The inter-trial interval was 15 min.

**Contextual and cued fear conditioning test**

The fear conditioning test was conducted using ImageJ FZ1 (O’Hara & Co., Ltd., Tokyo, Japan). The conditioning chamber was a square arena (10 cm × 10 cm × 10 cm) with clear Plexiglas walls and a metal grid floor connected to a circuit board that delivered electric shocks to the metal grid. A video camera was set in front of the cage to record the behavior. In the conditioning session, mice were individually placed into the conditioning chamber and allowed to explore freely at 3 min. After 3 min exploratory period, each mouse was exposed to two tone-footshock pairings (tone, 30 sec; footshock, 2 sec, 0.8 mA at the termination of the tone; separated by 1 min intertrial interval). One min after the second footshock, the mouse was returned to its home cage. 24 h after conditioning, the context-dependent test was performed, in which each mouse was placed back into the conditioning chamber, and the freezing response was measured for 6 min in the absence of the conditioned stimulus. 48 h after the footshock, each mouse was tested for auditory (tone) fear conditioning in a novel opaque chamber. Different environmental cues (e.g. light condition and background noise) were provided in the novel chamber. Mice were tested in the novel chamber for a 3 min baseline period (pre-tone) followed by another 3 min for the conditioning tone during which the tone was presented persistently for 3 min. Total freezing rate was measured as an index of fear memory. Motionless bouts lasting more than 2 s were considered as freeze.

**Apparatus for touchscreen-based visual discrimination (VD) learning and reversal learning**

Behavioral training was performed using the touchscreen chamber system (Phenosys, Berlin, Germany; Brain science Idea, Osaka, Japan). The touch screen monitor (5 × 4 inch) was on the front of the chamber and covered by a black plastic mask with 2 response windows (40 × 40 mm^2^) to prevent accidental touches. The nozzle for reward delivery was located on the opposite side of the touchscreen monitor. A reward was delivered via the nozzle using a peristaltic pump through a plastic window in the wall. The operant arena (5 cm × 18 cm × 20 cm × 20 cm) consisted of a perforated metal gird floor enclosed by 2 black plastic trapezoidal walls toward to the screen. The operant chamber was placed inside a sound- and light- attenuating box equipped with a fan to provide ventilation and mask background noise. The top of the chamber was covered with a transparent plastic lid.

**Touchscreen-based VD and reversal learning**

The protocol used was described in detail in previous studies (13, 14). Briefly, the battery touchscreen-based behavioral tasks were started with food and water restrictions to motivate mice to perform the task. Food and water restrictions were maintained throughout the touchscreen tasks, and the body weight of test mice was maintained at 85–90% of unrestricted animals. The pre-training includes habituation, initial touch, must touch, must initiate and punish correct. The criterion for successful learning was defined as 75% of correct responses for 2 consecutive sessions in pre-training.

In VD task, a pair of stimuli was presented simultaneously on the screen of pseudorandom locations. Touching the correct stimulus resulted in a milk liquid reward, touching the incorrect response resulted in a 5-second time-out punishment followed by a correction trial. In the correction trials, the pair of stimuli was repeatedly presented on the same screen until the mice make a correct response. The session finishes 60 min or 30 after trials are completed, whichever comes first. The total number of trials, correction trials and correction errors as well as the percentage of correct responses and the perseveration index (the average of sequential correction errors) in different training stages were evaluated. The criterion for successful learning of the VD task was defined as more than 80% of correct responses for 2 consecutive sessions.

The reversal learning is similar to the VD task described above, except that the correct and incorrect stimuli for reward were reversed. The criterion for successful learning of reversal learning was defined as more than 80% of correct responses for 2 consecutive days or completion of 20 sessions.

**Animals and behavioral rhythm measurement**

Spontaneous locomotor activities of male *Del(3.0Mb)/+* (n = 14) and WT (n = 13) mice were measured as described previously (15). Briefly, mice were housed individually in recording cages at the age of 13–16 weeks. Their movement was recorded every minute by an infrared thermal sensor. Behavioral activity rhythms were analyzed by Clock Lab (Actimetrics, IL, USA), and circadian periods were determined by a χ^2^-periodogram.

**Electrophysiology**

300-μm-thick prefrontal coronal slices were prepared from 2-week-old mice in the following cutting solution; 120 mM Choline Cl, 28 mM NaHCO_3_, 1.25 mM NaH_2_PO_4_, 2 mM KCl, 25 mM glucose, 1 mM CaCl_2_, 8 mM MgCl_2_, bubbled with 95 % O_2_ and 5 % CO_2_ with a vibratome slicer (Leica). The slices were incubated at room temperature for 30–45 min with artificial cerebrospinal fluid (ACSF) composed of 125 mM NaCl, 2.5 mM KCl, 2 mM CaCl_2_, 1 mM MgSO_4_, 1.25 mM NaH_2_PO_4_, 26 mM NaHCO_3_, and 20 mM glucose bubbled with 95 % O_2_ and 5 % CO_2_.

In the whole cell patch clamp recording, pyramidal neurons were identified in layer 2/3 of medial prefrontal cortex (mPFC) morphologically. The pipet resistance was 2.4-5 MΩ and it was filled with internal solution of 130 mM K D-gluconate, 6 mM KCl, 10 mM NaCl, 10 mM HEPES, 0.16 mM CaCl_2_, 2 mM MgCl_2_, 0.5 mM EGTA, 4 mM Na-ATP, and 0.4 mM Na-GTP (pH 7.3, adjusted with KOH) for miniature excitatory postsynaptic current (mEPSC). For miniature inhibitory postsynaptic current (mIPSC), the recording pipettes were filled with the following internal solution: 145 mM KCl, 10 mM HEPES, 10 mM EGTA, 0.16 mM CaCl_2_, 2 mM MgCl_2_, 5 mM Mg-ATP, and 0.2 mM Na-GTP (pH 7.2, adjusted with KOH). All of the recordings were performed at 30–32°C with EPC-10 amplifier (HEKA Elektronik, Lambrecht/Pfalz, Germany). The data was filtered at 2.9 kHz and digitized at 20 kHz. An access resistance of the recording pipettes was compensated by 70 % in the recording. The miniature synaptic responses were recorded at −70 mV in the presence of 0.5 μM tetrodotoxin (TTX) (Nacalai Tesque, Nagoya, Japan) with 0.1 mM picrotoxin (mEPSC) or 10 μM NBQX, 50 μM D-AP5 (mIPSC). Each synaptic response was detected by Mini Analysis program (Synaptosoft Inc., GA, USA) with eyes using the following criteria; over 5 pA, rise time < 3 ms. All of the experiments were performed without information about the genotypes.

**Visual evoked potentials**

Visual evoked potentials were recorded from 11-week-old WT (n = 8) and *Del(3.0Mb)/+* mice (n = 7). Under pentobarbital anesthesia (40-50 mg/kg, i.p.), a recording electrode (polyurethane-coated stainless-steel wire with 100 μm in diameter) was chronically implanted into the left visual cortex (2.2 mm lateral, 4.0 mm posterior to bregma, 400 μm ventral to the dura mater). A gold plating pin was positioned in the right frontal bone (0.5 mm lateral, 3.0 mm anterior to bregma) as a reference electrode. Electrodes and a stainless frame for awake head-fixed recording (12 mm × 19 mm, CF-10, Narishige Co. Ltd., Tokyo, Japan) were fixed to the skull with dental acrylic cement.

Visual stimuli were generated and given according to the previous report (Hamm et al., 2016). Briefly, we presented static full-field square-wave grating (100% contrast, 0.04 cycles/degree) on a monitor positioned 15 cm away from the right eye, roughly at 45° to the long axis of the animal. Stimuli were displayed for 500 ms, followed by an interstimulus interval of 1,000 ms of mean luminescence gray screen. A session was composed of 600 stimuli and lasted 15 min.

Four to five days after the implantation, LFP of the left visual cortex was recorded in head-fixed mice at sampling rate of 4 kHz with a 50-Hz hum filter. Signals were amplified (MEG1200, Nihon-koden, Tokyo, Japan) and digitized (PowerLab, AD instruments, Dunedin, New Zealand) for analysis. LFP data were processed using MATLAB. The data were resampled at frequency of 250 Hz and prescreened for excessive artifact (e.g. signal greater than 5 SDs).

**Supplemental Table 1.** sgRNA and single-stranded oligodeoxyribonucleotide (ssODN) sequences for generating *Del(3.0Mb)/+* mice.

| sgRNA | sequences (5'–3') | ssODN 3.0-Mb bridge (5'–3') |
| --- | --- | --- |
| Pi4ka sg1 | CAGGACTGGGACTCGAGACGGGG | GCCAGAGCCTGATGCTCCCTTGCAGGAGCACGGCCACCTCCATACTGATGCTGGCCTCCATCCAGGGGCTGATGAATGAGTGAGGAACTATTCATGGTTCTCTAGGCCTCAAGTAGAAAG |
| Pi4ka sg2 | ATGCGGCCCCACAGATCTGGAGG |  |
| Hira sg1 | GAGGAGGTCGCCTATTGTCCAGG |  |
| Hira sg2 | GGTAGAAGGAGTGGGCTAACAGG |  |

**Supplemental Table 2.** Primer sequences for quantitative RT-PCR.

| Gene | Forward | | Reverse | |
| --- | --- | --- | --- | --- |
| *Hprt* | GGTTAAGCAGTACAGCCCCA | | GTCTGGCCTGTATCCAACACT | |
| *Actb* | CCTTCTTGGGTATGGAATCCTGT | | TGGCATAGAGGTCTTTACGGATG | |
| *Pi4ka* | GGCTGGGAACCAGACATCAA | | CATCCATATAGGGGCGCACA | |
| *Serpind1* | GCCATTGACCTGTTCAAGCA | | GGGACGGTCGACAGTGAATC | |
| *Snap29* | AACCTAGATGAGCTGTCCGTG | | TGGTTGTCAGTCGGTCAAGG | |
| *Crkl* | ACGTGCTAGATAACCGGCTG | | TTTCAGCTGAGGCTGGGATG | |
| *Aifm3* | CTTGCCTGGAGGAACAATCG | | CATGGCAGTCCACAGATAGGG | |
| *Lztr1* | GGCTTACTGCAAGCAAAACCT | | GCAGCTTAGAGACCTTGGTGA | |
| *Thap7* | CTGGTGGGAATCAGTGGGTA | | TGTTCGACGCAACTTGGAGA | |
| *Lrrc74b* | TGACCTGGCAGGAGAGATACT | | CAGACCTCGCAGGTGATTCC | |
| *P2rx6* | GAAGTTCGCGCTCATCCCTA | | GGGGCTCTTGCCTCTTCATA | |
| *Slc7a4* | CTGGTCGGACTTGTCGTGT | | CTCCCGGACTGGTGATTGAC | |
| *Smpd4* | CCGGAGCTACGAAATCACCA | | TCTGGCCTGCAAATCTACGG | |
| *Ccdc74a* | GAGATCGAGCACCTGAAGCG | | GAGAGTTGGCGGACATCGTG | |
| *Med15* | CACCTATCGTGTCGCCAGTG | | ACCTTGCAGCACATTGGGTA | |
| *Klhl22* | CCCCACTCAAGAAGGAGGTAT | | TATGCCACGTGTTGCTTCCT | |
| *Scarf2* | GAAGGCGCCTCAACGTTTTT | | ATGGGCCACTACGACTTTGG | |
| *Car15* | CATGCGCAGGTAGTCCAGTT | | GGGGCGGAAATTACTCGTGA | |
| *Dgcr2* | TTCCATTTCCACGACCCTCC | | CTCAAAGGCATCGTCATCTGC | |
| *Tssk1* | CGGTCTGAGTCAAAACCCCA | | GGCTGCAAGAGGCTCACTAA | |
| *Dgcr14* | ACTTGGCCAGTCTTACTCCCA | | GAGGTGGGAAGATCGTGCTG | |
| *Slc25a1* | TGCAGCCAGTGTCTTTGGAA | | GTAGAATGCCTTTGGCCCCT | |
|  |  | | *(Table continued on next page)* | |
| *(Continued)* |  | |  | |
| *Dgcr6* | CGAACACCGAGTGCTCAGAC | | ATCCGATGTTCCATGGCCT | |
| *Prodh* | TTTATGCCCAAGGCGGGATT | | TTGGGGTACAGGAAGTCCCA | |
| *Rtn4r* | GAGCTTCCAGTCATGCCGAA | | GGTCCACGACATGAAGCTGT | |
| *Zdhhc8* | AGAAACCTCTGGACCTGGGA | | TGATAGGGCACTCTCAGCAC | |
| *Ranbp1* | CTTCCTAAATGCTGAGAATGCAC | | CCTCCCTCACTGAAAGGGC | |
| *Trmt2a* | TAAGGTGATTCTGGCCATCCG | | CCTGCAGAGGTCCACAAAGTT | |
| *Dgcr8* | GCGCGGGTGGTGTAAGAATAA | | TGCTGCTCTCACGACCATAC | |
| *Arvcf* | ACGAAGTCACGCTTCCAGTC | | TTGACTTCTCCCCATCAAGGC | |
| *Comt* | ATTGTGGCTACTCAGCCGTG | | CCCGATGAGGATGGAAACTTTG | |
| *Txnrd2* | GGATCAAGTGTGGGGCTTCA | | GCAACCAGTCACAGTAGGCT | |
| *Gnb1l* | TCCCTGCAGGTGAAGAAGACT | AACACACGGATGCGATGGTC | |  |
| *Gp1bb* | AGTGATGGAACAGCCCAGTC | TTTGGCAAAGTCGGGTGGTA | |  |
| *Sept5* | AACATGCTCATCCGCACTCA | CTGGGTGAGTTTGCTGGTCA | |  |
| *Cldn5* | GTTAAGGCACGGGTAGCACT | TACTTCTGTGACACCGGCAC | |  |
| *Cdc45* | GAGGGCACTCCAGATGTCAC | GCATCGTCGATTCTTTGTCGAG | |  |
| *Ufd1l* | TTGAAGAGGATGAAGCTGGAGG | CAGTAACTGTAAGCCAGGTGC | |  |
| *Mrpl40* | GCAGCAAAGGATCGCTTGAA | CTCCTGAGGTCGCTGTCTTG | |  |
| *Hira* | CCTCCAACTCTGGAAGGCAAG | AGGAGGAGCCAGTGACGATA | |  |

**Supplemental Table 3.** Statistical analysis related to Figure 2–5 and Supplemental Figure 4–5.

| Figure 2a | Two-tailed Welch's *t*-test. *t*_16.68_ = 2.393. **p* = 0.0288.  WT: n = 13, *Del(3.0Mb)/+*: n = 9. |
| --- | --- |
| Figure 2b | Two-tailed Welch's *t*-test. *t*_18.56_ = 2.531. **p* = 0.0206.  WT: n = 13, *Del(3.0Mb)/+*: n = 9. |
| Figure 2c | Two-tailed Welch's *t*-test. *t*_10.36_ = 0.996. *p* = 0.3420.  WT: n = 13, *Del(3.0Mb)/+*: n = 9. |
| Figure 2d | Two-way repeated measures ANOVA.  Genotype: *F*_1,28_ = 11.07, ***p* = 0.0025;  Prepulse intensity: *F*_3,84_ = 83.06, *p* < 0.0001;  Genotype × Prepulse intensity interaction: *F*_3,84_ = 0.5792, *p* = 0.6303.  WT: n = 15, *Del(3.0Mb)/+*: n = 15. |
| Figure 2e | Two-tailed Welch's *t*-test. *t*_22.96_ = 2.101. **p* = 0.0468.  WT: n = 15, *Del(3.0Mb)/+*: n = 15. |
| Figure 2f | Two-way repeated measures ANOVA.  Genotype: *F*_1,20_ = 0.0569, *p* = 0.8139;  Prepulse intensity: *F*_1,20_ = 0.2033, *p* = 0.6570;  Genotype × Prepulse intensity interaction: *F*_1,20_ = 0.6502, *p* = 0.4295.  WT: n = 13, *Del(3.0Mb)/+*: n = 9. |
| Figure 2g | 1–4 trial: Two-way repeated measures ANOVA.  Genotype: *F*_1,20_ = 0.2479, *p* = 0.6240; Trial: *F*_3,60_ = 48.34, *p* < 0.0001; Genotype × Trial interaction: *F*_3,60_ = 0.0582, *p* = 0.9814.  5 trial: Two-tailed Welch's *t*-test. *t*_16.68_ = 2.161. **p* = 0.0456. WT: n = 13, *Del(3.0Mb)/+*: n = 9. |
| Figure 2h | Two-tailed Welch's *t*-test. Context: *t*_31.45_ = 2.014. *p* = 0.0526. Pre-tone: *t*_39.72_ = 0.4907. *p* = 0.6263. Tone: *t*_37.63_ = 5.367. ****p* < 0.001. WT: n = 24, *Del(3.0Mb)/+*: n = 20. |
| Figure 3b | Two-tailed Welch's *t*-test. *t*_10.49_ = 0.2675. *p* = 0.7943.  WT: n = 7, *Del(3.0Mb)/+*: n = 6. |
| Figure 3c | Two-tailed Welch's *t*-test. *t*_8.955_ = 4.136. ***p* = 0.0025.  WT: n = 7, *Del(3.0Mb)/+*: n = 6. |

*(Table continued on next page)*

*(Continued)*

| Figure 3d | Two-way repeated measures ANOVA.  Genotype: *F*_1,11_ = 8.242, **p* = 0.0152; Session: *F*_3,33_ = 20.99, *p* < 0.0001;  Genotype × Session interaction: *F*_3,33_ = 2.073, *p* = 0.1228.  WT: n = 7, *Del(3.0Mb)/+*: n = 6. |
| --- | --- |
| Figure 3e | Two-tailed Welch's *t*-test. R early: *t*_6.376_ = 0.9963. *p* = 0.3554.  R middle: *t*_10.25_ = 0.3115. *p* = 0.7616. R late: *t*_10.99_ = 0.4749. *p* = 0.6442. WT: n = 7, *Del(3.0Mb)/+*: n = 6. |
| Figure 3f | Two-tailed Welch's *t*-test. R early: *t*_6.302_ = 0.8051. *p* = 0.4501.  R middle: *t*_10.98_ = 0.3581. *p* = 0.7271. R late: *t*_10.97_ = 0.7466. *p* = 0.4710. WT: n = 7, *Del(3.0Mb)/+*: n = 6. |
| Figure 3g | Two-tailed Welch's *t*-test. *t*_25.59_ = 1.179. *p* = 0.2494.  WT: n = 20, *Del(3.0Mb)/+*: n = 20. |
| Figure 3h | Two-tailed Welch's *t*-test. *t*_37.72_ = 0.7169. *p* = 0.4778.  WT: n = 20, *Del(3.0Mb)/+*: n = 20. |
| Figure 3i | Two-tailed Welch's *t*-test. *t*_37_ = 0.3944. *p* = 0.6955.  WT: n = 19, *Del(3.0Mb)/+*: n = 20. |
| Figure 3j | Two-tailed Welch's *t*-test. *t*_25.64_ = 0.9568. *p* = 0.3476.  WT: n = 19, *Del(3.0Mb)/+*: n = 20. |
| Figure 4d | Two-tailed Welch's *t*-test. N1: *t*_12.91_ = 0.7473. *p* = 0.4683.  P1: *t*_11.84_ = 2.869. **p* = 0.0143. N2: *t*_12.18_ = 2.876. **p* = 0.0138. WT: n = 8, *Del(3.0Mb)/+*: n = 7. |
| Figure 4e | Two-tailed Welch's *t*-test. N1: *t*_6.372_ = 2.342. *p* = 0.0552.  P1: *t*_7.194_ = 2.140. *p* = 0.0686. N2: *t*_12.99_ = 0.9256. *p* = 0.3715. WT: n = 8, *Del(3.0Mb)/+*: n = 7. |

*(Table continued on next page)*

*(Continued)*

| Figure 5a | Two-tailed Welch's *t*-test. *t*_23.48_ = 1.991. *p* = 0.0583.  WT: n = 13, *Del(3.0Mb)/+*: n = 14. |
| --- | --- |
| Figure 5b | Two-tailed Welch's *t*-test.  Phase delay: *t*_24.97_ = 0.5221. *p* = 0.6062. WT: n = 13, *Del(3.0Mb)/+*: n = 14. Phase advance: *t*_20.88_ = 1.129. *p* = 0.2715. WT: n = 13, *Del(3.0Mb)/+*: n = 13. |
| Figure 5c | Two-tailed Welch's *t*-test.  Phase shift (left panel): *t*_15.3_ = 2.616. **p* = 0.0192.  WT: n = 13, *Del(3.0Mb)/+*: n = 13. Phase shift (right panel): *t*_21.42_ = 0.8958. *p* = 0.3803.  WT: n = 12, *Del(3.0Mb)/+*: n = 13. |
| Figure 5d | Sidak's multiple comparison after two-way repeated measure ANOVA. Genotype: *F*_1,25_ = 0.3379, *p* = 0.5663; Time: *F*_23,575_ = 33.96, *p* < 0.0001;  Genotype × Time interaction: *F*_23,575_ = 2.19, *p* = 0.0012. WT vs. *Del(3.0Mb)/+*; 16 (h): **p* = 0.0268.  WT: n = 14, *Del(3.0Mb)/+*: n = 13. |
| Figure 5e | Sidak's multiple comparison after two-way repeated measure ANOVA. Genotype: *F*_1,25_ = 0.2003, *p* = 0.6583; Time: *F*_23,575_ = 82.19, *p* < 0.0001;  Genotype × Time interaction: *F*_23,575_ = 1.86, *p* = 0.0090. WT vs. *Del(3.0Mb)/+*; 22 (h): **p* = 0.0337, 0 (h): **p* = 0.0411.  WT: n = 14, *Del(3.0Mb)/+*: n = 13. |
| Supplemental Figure 4a | Cumulative counts at 120 min: Two-tailed Welch's *t*-test.  *t*_25.9_ = 1.295. *p* = 0.2067. WT: n = 15, *Del(3.0Mb)/+*: n = 15. |
| Supplemental Figure 4b | Two-tailed Welch's *t*-test. 0-1 h: *t*_27.32_ = 1.405. *p* = 0.1714.  1-2 h: *t*_24.92_ = 0.8106. *p* = 0.4253. Total: *t*_25.9_ = 1.295. *p* = 0.2067. WT: n = 15, *Del(3.0Mb)/+*: n = 15. |
| Supplemental Figure 4c | Two-way repeated measures ANOVA.  Genotype: *F*_1,28_ = 1.677, *p* = 0.2059; Time: *F*_23,644_ = 47.54, *p* < 0.0001;  Genotype × Time interaction: *F*_23,644_ = 1.235, *p* = 0.2063.  WT: n = 15, *Del(3.0Mb)/+*: n = 15. |

*(Table continued on next page)*

*(Continued)*

| Supplemental Figure 4d | 1-3 day: Two-way repeated measures ANOVA.  Genotype: *F*_1,28_ = 0.8122, *p* = 0.3751; Day: *F*_2,56_ = 86.71, *p* < 0.0001;  Genotype × Day interaction: *F*_2,56_ = 1.27, *p* = 0.2889.  Test: Two-tailed Welch's *t*-test. *t*_25.39_ = 1.131. *p* = 0.2688. WT: n = 15, *Del(3.0Mb)/+*: n = 15. |
| --- | --- |
| Supplemental Figure 4e | Two-tailed Welch's *t*-test. *t*_17.76_ = 0.6585. *p* = 0.5187.  WT: n = 15, *Del(3.0Mb)/+*: n = 15. |
| Supplemental Figure 4f | Two-tailed Welch's *t*-test. *t*_23.77_ = 0.0406. *p* = 0.9680.  WT: n = 15, *Del(3.0Mb)/+*: n = 15. |
| Supplemental Figure 4g | Two-tailed Welch's *t*-test.  Empty: *t*_27.59_ = 0.3894. *p* = 0.7000. Stranger: *t*_27.76_ = 0.02859. *p* = 0.9774.  WT: n = 15, *Del(3.0Mb)/+*: n = 15. |
| Supplemental Figure 4h | Two-tailed Welch's *t*-test.  Familiar: *t*_28_ = 0.3322. *p* = 0.7422. Stranger: *t*_27.8_ = 0.1378. *p* = 0.8914.  WT: n = 15, *Del(3.0Mb)/+*: n = 15. |
| Supplemental Figure 5a | Two-tailed Welch's *t*-test. *t*_27.81_ = 2.958. ***p* = 0.0063.  WT: n = 15, *Del(3.0Mb)/+*: n = 15. |
| Supplemental Figure 5b | Two-tailed Welch's *t*-test. *t*_27.94_ = 2.27. **p* = 0.0311.  WT: n = 15, *Del(3.0Mb)/+*: n = 15. |
| Supplemental Figure 5c | Two-tailed Welch's *t*-test. *t*_26.66_ = 0.09187. *p* = 0.9275.  WT: n = 15, *Del(3.0Mb)/+*: n = 15. |
| Supplemental Figure 5d | Two-tailed Welch's *t*-test. Familiar: *t*_27.83_ = 1.545. *p* = 0.1337 Novel: *t*_26.43_ = 0.1498. p = 0.8820.  WT: n = 15, *Del(3.0Mb)/+*: n = 15. |
| Supplemental Figure 5e | Two-tailed Welch's *t*-test. *t*_26.97_ = 1.513. *p* = 0.1419.  WT: n = 15, *Del(3.0Mb)/+*: n = 15. |

**Supplemental Table 4.** Efficiencies of generating *Del(3.0Mb)/+* mice.

| Deletion model | Embryos  injected^a^ | Transferred^b^  (%: b/a) | Pups born^c^  (%: c/b) | Weaning^d^  (%: d/c) | Desired mutant^e^  (%: e/d) |
| --- | --- | --- | --- | --- | --- |
| *Del(3.0Mb)/+* | 1119 | 644 (57.6) | 95 (14.8) | 74 (77.9) | 4 (5.4) |

**Supplemental Table 5.** Neonatal mortality rate of *Del(3.0Mb)/+* mice before weaning.

| Number of pups | | Number of deaths | | Weaning | |
| --- | --- | --- | --- | --- | --- |
| WT^a^ | *Del(3.0Mb)/+*^b^ | WT^c^  (%: c/a) | *Del(3.0Mb)/+*^d^  (%: d/b) | WT^e^  (%: e/a) | *Del(3.0Mb)/+*^f^  (%: f/b) |
| 265 | 211 | 77 (29.1) | 149 (70.6) | 188 (70.9) | 62 (29.4) |

**Supplemental Table 6.** Appearance rate of cardiovascular and thymic abnormalities observed in *Del(3.0Mb)/+* embryos (E18.5).

| Genotype | Number of embryos | Phenotype | | |
| --- | --- | --- | --- | --- |
|  |  | IAA | ARSA | Thymus |
| WT | 18 | 0 (0%) | 0 (0%) | 0 (0%) |
| *Del(3.0Mb)/+* | 15 | 2 (13.3%) | 1 (6.7%) | 7 (46.7%) |

IAA, interrupted aortic arch; ARSA, aberrant right subclavian artery; Thymus, thymic hypoplasia.

**Supplemental Table 7.** Comparison of 22q11.2DS-related phenotypes in model mice.

(*Table continued on next page*)

*(Continued)*

↑, increase or improved behavior; ↓, decrease or impaired behavior; n.c., not changed; N/A, not applicable.

Total, total distance moved; Margin, distance moved in margin zone; Basal SI, basal social interaction; PPI, prepulse inhibition.

Parenthesis in the fear conditioning column expresses the time that has elapsed from the fear conditioning phase.

*Deletion length varies from individual patients.


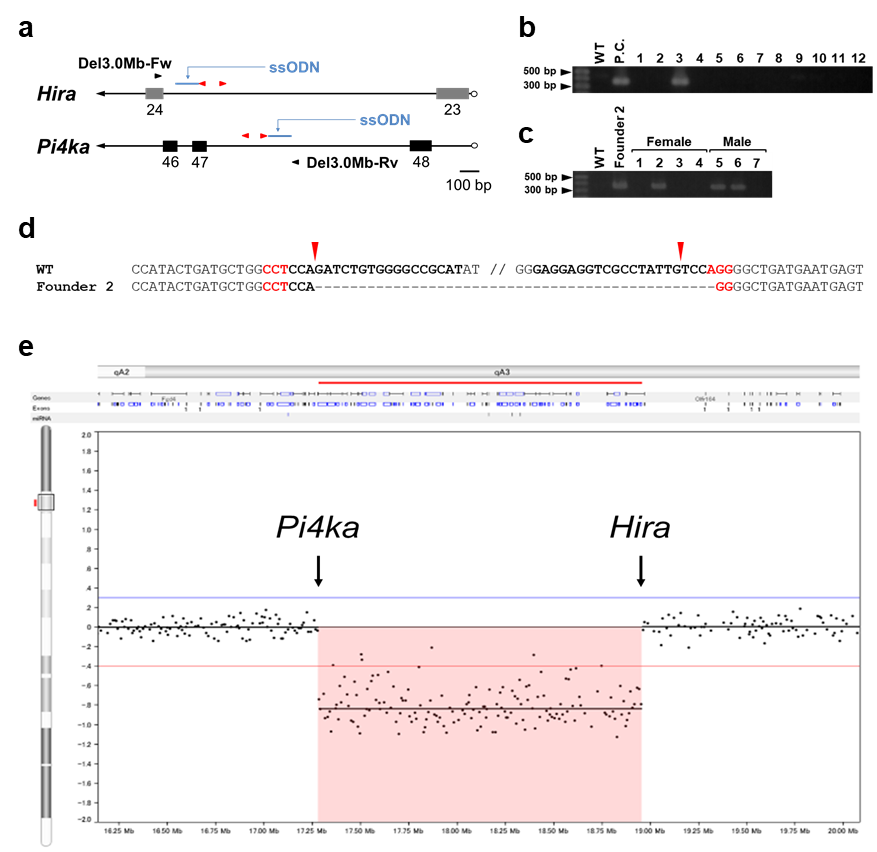


**Supplemental Figure 1.**

Generation of *Del(3.0Mb)/+* mouse using CRISPR/Cas9 system. **a,** Schematic illustration showing the CRISPR/Cas9 sgRNA sites in *Pi4ka* intron 47 and *Hira* intron 23 (red arrowheads), the genotyping primer sites (black arrowheads) and the bridging oligonucleotides (blue). Black and gray squares indicate exons. **b,** A representative result of genotyping PCR analysis of founder candidates. P.C., positive control. **c,** Genotyping analysis of N1 offspring of *Del(3.0Mb)/+* founder × wild type crossing. **d,** The nucleotide sequence analysis of a PCR-amplified fragment around deletion junction in *Del(3.0Mb)/+* founder. sgRNA sites are indicated in bold and PAM sites in red. The expected sgRNA-guided cutting sites are indicated by red arrowheads. **e,** Array CGH profile of chromosome 16qA13 from N1 *Del(3.0Mb)/+* mouse showing the decrease in copy number of targeting region (pink). Array CGH data are shown in an engaged view. Black arrows indicate the both ends of deleted region (*Pi4ka* and *Hira*).


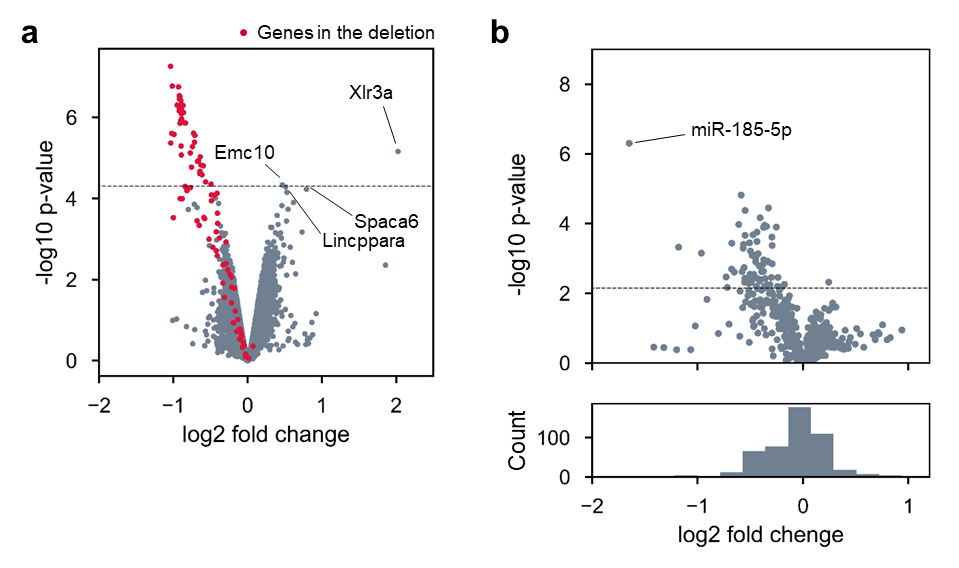


**Supplemental Figure 2.**

mRNA and miRNA expression in the frontal cortex of *Del(3.0Mb)/+* mice. **a,** Volcano plot of the mRNA microarray analysis data of the frontal cortex. The x-axis shows log_2_ of the fold change (*Del(3.0Mb)/+* vs. WT). The genes in the deleted region are depicted in red. The horizontal dashed line indicates the *p*-value of FDR = 0.05. **b,** The microarray analysis of miRNA expression in the frontal cortex. Top: Volcano plot of the microarray analysis data. The horizontal dashed line indicates the *p*-value of FDR = 0.05. Bottom: Histogram shows the distribution of the probesets across the fold change.


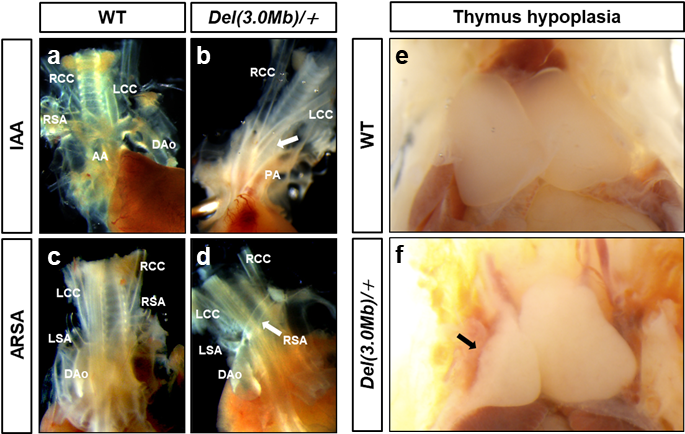


**Supplemental Figure 3.**

Examples of cardiovascular and thymic defects in *Del(3.0Mb)/+* E18.5 embryos. **a,** Normal anatomy in a WT embryo. Observation of the heart from the ventral. **b,** Interrupted aortic arch (IAA) in a *Del(3.0Mb)/+* embryo. White arrow shows the point of the anomaly. **c,** Normal anatomy in a WT embryo. Observation of the heart from the dorsal. **d,** Aberrant right subclavian artery (ARSA) from the descending aorta in a *Del(3.0Mb)/+* embryo. White arrow shows the abnormal blood vessel. AA, aortic arch; DAo, descending aorta; LCC, left common carotid artery; LSA, left subclavian artery; RCC, right common carotid artery; RSA, right subclavian artery. **e, f,** Representative thymi from a WT control embryo (e) and a *Del(3.0Mb)/+* embryo (f). Black arrow shows thymic hypoplasia in a *Del(3.0Mb)/+* embryo.


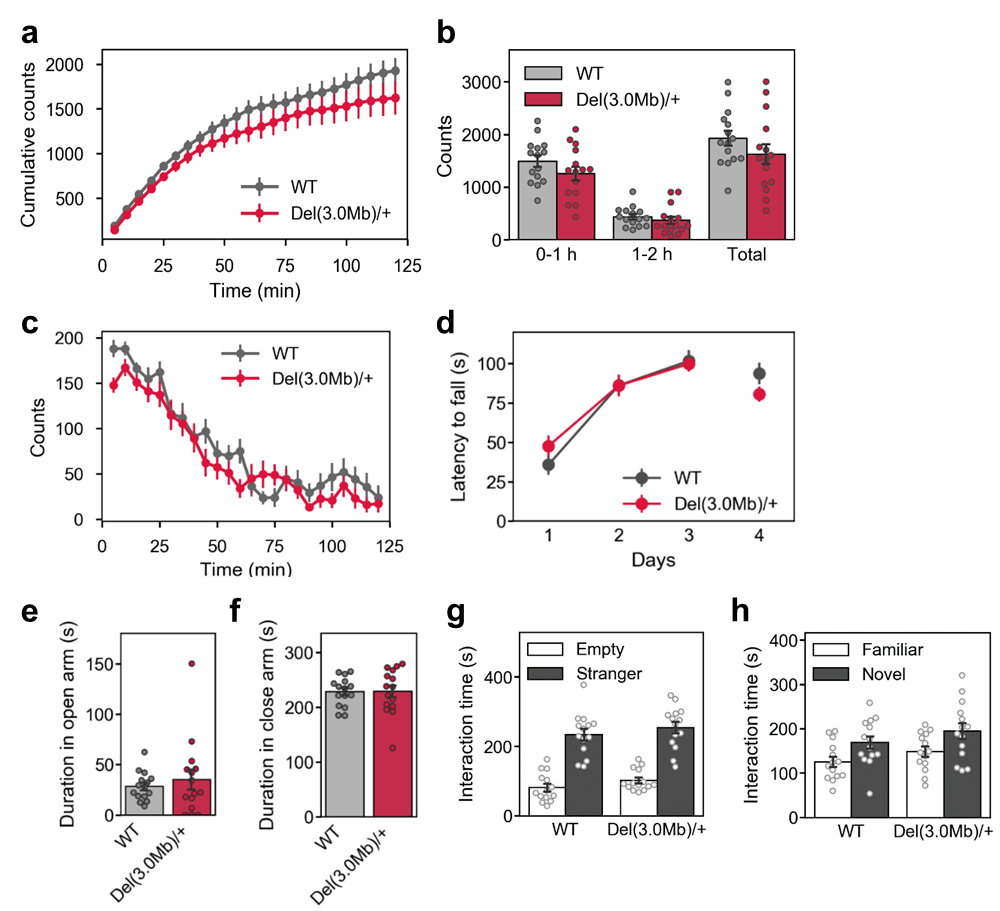


**Supplemental Figure 4.**

Locomotor activity and anxiety-like behavior of *Del(3.0Mb)/+* mice. **a–d,** Locomotor activity in novel environment. **a,** The cumulative count curves of the number of moving actions in 120 min. **b,** The counts averaged in every 1 h. **c,** The counts averaged in every 5 min. **d,** Latency to fall in rotarod test. **e, f,** Elevated plus maze test. Time spent in open arm (e) and in closed arm (f). **g, h,** Three-chamber sociability and social novelty test. **g,** Interaction time of the sociability test. **h,** interaction time of social novelty test. Data are expressed as mean ± SEM (WT, n = 15; *Del(3.0Mb)/+*, n = 15).


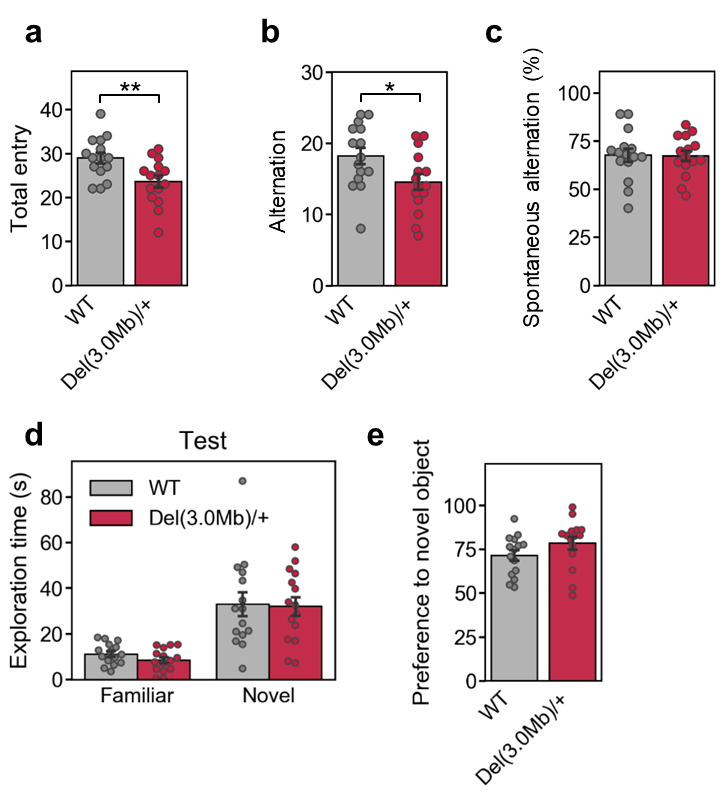


**Supplemental Figure 5.**

Measurements of cognitive function by Y-maze and novel object recognition test in *Del(3.0Mb)/+* mice. **a–c,** Y-maze test. **a,** The number of arm entries. **b, c,** The number and percentage of spontaneous alternation. **d, e,** Novel object recognition test. **d,** The data of exploration time to familiar and novel objects. **e,** The percentage of exploratory preference to each of objects. All data are expressed as means ± SEM (WT, n = 15; *Del(3.0Mb)/+*, n = 15). **p* < 0.05. ***p* < 0.01.

**Supplemental Refferences**

1. Naito, Y., Hino, K., Bono, H. & Ui-Tei, K. CRISPRdirect: software for designing CRISPR/Cas guide RNA with reduced off-target sites. *Bioinformatics*. 31, 1120–1123 (2015).

2. Nakao, H. et al. A possible aid in targeted insertion of large DNA elements by CRISPR/Cas in mouse zygotes. *Genesis*. 54, 65–77 (2016).

3. Kushima, I. et al. Comparative analyses of copy-number variation in autism spectrum disorder and schizophrenia reveal etiological overlap and biological insights. *Cell Rep*. 24, 2838–2856 (2018).

4. Livak, K.J. & Schmittgen, T.D. Analysis of relative gene expression data using real-time quantitative PCR and the 2 (-Delta Delta C (T)) method. *Methods*. 25:402-408 (2001).

5. Alkam, T., Nitta, A., Mizoguchi, H., Itoh, A. & Nabeshima, T. A natural scavenger of peroxynitrites, rosmarinic acid, protects against impairment of memory induced by A beta (25-35). *Behav. Brain Res*. 180, 139–145 (2007).

6. Miyamoto, Y. et al. Lower sensitivity to stress and altered monoaminergic neuronal function in mice lacking the NMDA receptor epsilon 4 subunit. *J. Neurosci*. 22, 2335–2342 (2002).

7. Kamei, H. et al. Repeated methamphetamine treatment impairs recognition memory through a failure of novelty-induced ERK1/2 activation in the prefrontal cortex of mice. *Biol. Psychiatry*. 59, 75–84 (2006).

8. Nagai, T. et al. Aripiprazole ameliorates phencyclidine-induced impairment of recognition memory through dopamine D-1 and serotonin 5-HT1A receptors. *Psychopharmacology*. 202, 315–328 (2009).

9. Moy, S.S. et al. Sociability and preference for social novelty in five inbred strains: an approach to assess autistic-like behavior in mice. *Genes Brain. Behav*. 3, 287–302 (2004).

10. Takahashi, K. et al. Neural circuits containing pallidotegmental GABAergic neurons are involved in the prepulse inhibition of the startle reflex in mice. *Biol. Psychiatry*. 62, 148–157 (2007).

11. Arai, S. et al. Involvement of pallidotegmental neurons in methamphetamine- and MK-801-induced impairment of prepulse inhibition of the acoustic startle reflex in mice: reversal by GABA(B) receptor agonist baclofen. *Neuropsychopharmacology*. 33, 3164–3175 (2008).

12. Aubert, L., Reiss, D. & Ouagazzal, A.M. Auditory and visual prepulse inhibition in mice: parametric analysis and strain comparisons. *Genes Brain. Behav*. 5, 423–431 (2006).

13. Horner, A.E. et al. The touchscreen operant platform for testing learning and memory in rats and mice. *Nat. Protoc*. 8, 1961–1984 (2013).

14. Wulaer, B. et al. Repetitive and compulsive-like behaviors lead to cognitive dysfunction in Disc1(∆ 2-3/∆ 2-3) mice. *Genes Brain. Behav*. 17, e12478 (2018).

15. Yoshitane, H. et al. JNK regulates the photic response of the mammalian circadian clock. *Embo Rep*. 13, 455–461 (2012).

16. Nilsson, S.R. et al. Assessing the cognitive translational potential of a mouse model of the 22q11.2 microdeletion syndrome. *Cereb. Cortex*. 26, 3991–4003 (2016).

17. Didriksen, M. et al. Persistent gating deficit and increased sensitivity to NMDA receptor antagonism after puberty in a new mouse model of the human 22q11.2 microdeletion syndrome: a study in male mice. *J. Psychiatry. Neurosci*. 42, 48–58 (2017).

18. Stark, K.L. et al. Altered brain microRNA biogenesis contributes to phenotypic deficits in a 22q11-deletion mouse model. *Nat. Genet*. 40, 751–760 (2008).

19. Fenelon, K. et al. The pattern of cortical dysfunction in a mouse model of a schizophrenia-related microdeletion. *J. Neurosci*. 33, 14825–14839 (2013).

20. Piskorowski, R.A. et al. Age-dependent specific changes in area CA2 of the hippocampus and social memory deficit in a mouse model of the 22q11.2 deletion syndrome. *Neuron*. 89, 163–176 (2016).

21. Diamantopoulou, A. et al. Loss-of-function mutation in Mirta22/Emc10 rescues specific schizophrenia-related phenotypes in a mouse model of the 22q11.2 deletion. *Pro. Natl. Acad. Sci. USA*. 114, e6127–e6136 (2017).

22. Meechan, D.W. et al. Cognitive ability is associated with altered medial frontal cortical circuits in the Lgdel mouse model of 22q11.2DS. *Cereb. Cortex*. 25, 1143–1151 (2015).

23. Marissal, T. et al. Restoring wild-type-like CA1 network dynamics and behavior during adulthood in a mouse model of schizophrenia. *Nat. Neurosci*. 21, 1412–1420 (2018).

24. Paylor, R. et al. Mice deleted for the DiGeorge/velocardiofacial syndrome region have shizophrenia-related behaviour and learning and memory impairments. *Hum. Mol. Genet*. 10, 2645–2650 (2001).

25. Paylor, R. et al. Tbx1 haploinsufficiency is linked to behavioral disorders in mice and humans: implications for 22q11 deletion syndrome. *Pro. Natl. Acad. Sci. USA*. 103, 7729–7734 (2006).

26. Eom, T.Y., Bayazitov, I.T., Anderson, K., Yu, J. & Zakharenko, S.S. Schizophrenia-related microdeletion impairs emotional memory through microRNA-dependent disruption of thalamic inputs to the amygdala. *Cell Rep*. 19, 1532–1544 (2017).

27. Sumitomo, A. et al. A mouse model of 22q11.2 deletions: Molecular and behavioral signatures of Parkinson's disease and schizophrenia. *Sci. Adv*. 4, eaar6637 (2018).

28. Schneider, M. et al. Psychiatric disorders from childhood to adulthood in 22q11.2 deletion syndrome: results from the international consortium on brain and behavior in 22q11.2 deletion syndrome. *Am. J. Psychiatry*. 171, 627–639 (2014).

29. Fung, W.L. et al. Elevated prevalence of generalized anxiety disorder in adults with 22q11.2 deletion syndrome. *Am. J. Psychiatry*. 167, 998–998 (2010).

30. Wong, L.M., Riggins, T., Harvey, D., Cabaral, M. & Simon, T.J. Children with chromosome 22q11.2 deletion syndrome exhibit impaired spatial working Memory. *Am. J. Intellect. Dev. Disabil*. 119, 115–132 (2014).

31. McCabe, K.L. et al. Visual perception and processing in children with 22q11.2 deletion syndrome: associations with social cognition measures of face identity and emotion recognition. *J. Neurodev. Disord*. 8, 30 (2016).

32. Fine, S.E. et al. Autism spectrum disorders and symptoms in children with molecularly confirmed 22q11.2 deletion syndrome. *J. Autism. Dev. Disord*. 35, 461–470 (2005).

33. Sobin, C., Kiley-Brabeck, K. & Karayiorgou, M. Lower prepulse inhibition in children with the 22q11 deletion syndrome. *Am. J. Psychiatry*. 162, 1090–1099 (2005).

34. Sobin, C., Kiley-Brabeck, K. & Karayiorgou, M. Associations between prepulse inhibition and executive visual attention in children with the 22q11 deletion syndrome. *Mol. Psychiatry*. 10, 553–562 (2005).

35. Cunningham, A.C. et al. Developmental coordination disorder, psychopathology and IQ in 22q11.2 deletion syndrome. *Br. J. Psychiatry*. 212, 27–33 (2018).

36. Debbane, M., Glaser, B. & Eliez, S. Encoding and retrieval processes in velo-cardio-facial syndrome (VCFS). *Neuropsychology*. 22, 226–234 (2008).

37. Shapiro, H.M., Tassone, F., Choudhary, N.S. & Simon, T.J. The development of cognitive control in children with chromosome 22q11.2 deletion syndrome. *Front. Psychol*. 5, 556 (2014).
